# Mesostructured Silica-Coated Magnetic Nanoparticles to Extract Six Opium Alkaloids in Poppy Seeds Prior to Ultra-High-Performance Liquid Chromatography-Tandem Mass Spectrometry Analysis

**DOI:** 10.3390/foods10071587

**Published:** 2021-07-08

**Authors:** Gema Casado-Hidalgo, Damián Pérez-Quintanilla, Sonia Morante-Zarcero, Isabel Sierra

**Affiliations:** Departamento de Tecnología Química y Ambiental, E.S.C.E.T., Universidad Rey Juan Carlos, C/Tulipán s/n, Móstoles, 28933 Madrid, Spain; gema.casado@urjc.es (G.C.-H.); damian.perez@urjc.es (D.P.-Q.); sonia.morante@urjc.es (S.M.-Z.)

**Keywords:** opium alkaloids, poppy seeds, *Papaver somniferum*, mesostructured silica, magnetic solid-phase extraction, liquid chromatography-tandem mass spectrometry

## Abstract

In recent years, health authorities have become increasingly concerned about preventing consumer exposure to opium alkaloids present in *Papaver somniferum* L. poppy seeds. In this study, a simple, rapid and efficient method has been optimised to determine all main opioids in poppy seeds (morphine, codeine, thebaine, papaverine, noscapine and oripavine) by UHPLC-QqQ-MS/MS. For this purpose, solid-liquid extraction (SLE) of samples was optimised and six magnetic adsorbent materials with a core of Fe_3_O_4_ coated with amorphous and mesostructured silica, both functionalised with octadecyl-silane or octyl-silane were characterised and evaluated for magnetic solid-phase extraction (MSPE). The material with the best results was non-functionalised mesostructured silica and, with it, the MSPE procedure was optimised. This method was validated and used to quantify six opioids in 14 edible seed samples (eleven poppy seeds and three seed mixes). Considerable amounts were found (1.5–249.0 mg/kg morphine, <0.2 µg/kg–45.8 mg/kg codeine, <2.4 µg/kg–136.2 mg/kg thebaine, <0.2 µg/kg–27.1 mg/kg papaverine, <0.2 µg/kg–108.7 mg/kg noscapine and <240 µg/kg–33.4 mg/kg oripavine), exceeding maximum limits established in some EU countries and the reference level of morphine in the EU. Furthermore, in some commercial samples for human consumption, inadequate labelling was found because significant amounts of alkaloids were detected even though *Papaver rhoeas* L. seeds were declared on the product label.

## 1. Introduction

The seeds of the opium poppy, *Papaver somniferum* L., are widely used in the preparation of food products such as bakery, buns, yoghurt, snacks or tea [1,2,3]. Although *Papaver somniferum* L. poppy seeds hardly contain any opium alkaloid, they can be contaminated with latex by poor harvesting practices or insect damage. The main opium alkaloids that may be present are morphine, codeine, papaverine, thebaine, noscapine and oripavine. However, most of the previously published studies have focused mainly on morphine and codeine, without paying attention to the other alkaloids for which concentrations may also be relatively high and of concern due to their possible high levels of toxicity [4]. In addition, another problem observed at the commercial level is that, in most cases, only the poppy seed is indicated on the label and the botanical name is not considered. This is an important aspect because *Papaver rhoeas* L. seeds (corn poppy seeds) do not contain any opium alkaloids unlike *Papaver somniferum* L. [5]. If food products are made from contaminated seeds, they may result in adverse health effects, especially in babies, infants, the elderly and people with severe health issues [6]. Moreover, their consumption can produce considerable amounts of opium alkaloids in biological samples such as blood, urine and oral fluid, which is enough to cause false positive drug abuse testing [7,8,9,10].

Nowadays, there is no harmonised European legislation on opium alkaloids in poppy seeds for food purposes and each country is carrying out different actions [4]. For example, only poppy seeds can be used in the production of bakery products in Belgium [11]. In Austria, a classification of varieties with low morphine content for food use has been established [12]. In Germany, a maximum limit of 4 mg/kg for morphine in poppy seed for use in food has been established and, in Hungary, limits of 30 mg/kg for morphine, 20 mg/kg for noscapine, 40 mg/kg for the sum of morphine and noscapine, 20 mg/kg for thebaine and 20 mg/kg for codeine are set [11]. Due to the absence of harmonised legislation among the EU Member States, a considerably high number of RASFF (Rapid Alert System for Food and Feed) health alerts have been generated in recent years [4]. For this reason, a reference level of 10 mg/kg morphine in poppy seeds for direct human consumption has been established in the EU. This level is not a maximum limit, but an agreement between EU Member States in November 2016 [13] until the Commission establishes that new risk management measures concerning the presence of opium alkaloids in food are necessary [5]. In addition, the European Food Safety Authority (EFSA) in 2011 carried out a risk assessment and established an acute reference dose (ARfD) of 10 µg morphine/kg body weight (b.w). In 2014, the European Commission published a set of recommendations for good agricultural and seed processing practices to reduce the morphine content in poppy seeds [14]. In 2018, a new EFSA opinion was published and its main conclusion was that more studies are needed to determine the presence of the six main opium alkaloids in edible seeds available on the market to evaluate the real exposure of consumers to all of these toxins and to create a harmonised legislation [15].

In order to carry out these studies, it is necessary to develop methods in food matrices that are effective, simple and rapid. Some of these alkaloids are found at ultra-trace levels in very complex matrices. For this reason, a selective and sensitive analytical technique is required. The most commonly used is chromatography, such as gas chromatography-coupled mass spectrometry (GC-MS) [10,16]. However, the costly step of sample derivatising has resulted in the increased use of (ultra)high-performance liquid chromatography ((U)HPLC). Multiple detectors can be used, such as the diode array [17] or ultraviolet [18], but the preferred technique for analysis of opium alkaloids is (U)HPLC coupled to triple quadrupole mass spectrometry ((U)HPLC-QqQ-MS/MS) with multiple reaction monitoring (MRM). However, to avoid co-eluted endogenous matrix components and to reduce the matrix effect that can be produced in MS detection, it is very important to do an adequate sample treatment in order to minimise the possibility of false results. Traditional procedures that are commonly used include solid-liquid extraction (SLE) with organic solvents [1,6] or solid-phase extraction (SPE) to perform sample purification [19,20]. The current trend in sample preparation involves the adoption of more automated, simple, fast and environmentally friendly approaches, mainly by integrating new adsorbent materials in the purification stage by means of microextraction techniques [4,21]. A powerful new purification alternative is solid-phase magnetic extraction (MSPE) that is faster and simpler. MSPE consists of dispersing the magnetic material in a solution with the sample for a few minutes. Once it is in equilibrium, it is recovered with the help of a magnetic field and, finally, the analytes are desorbed which can avoid tedious filtration, centrifugation or sedimentation steps [22]. Adsorbent material type is an important parameter in MSPE procedure because it will determine the ability to purify the samples. Until now, Fe_3_O_4_ particles covered with amorphous silica are the most used as a magnetic adsorbent, which can be functionalised with different organic groups as natural polymers or graphene [22,23,24,25]. However, the use of mesostructured silica is increasingly important because of their larger surface area, which can bind more of the functional groups and their uniform porous structure can facilitate access to the analytes [26,27]. However, MSPE procedures with this type of adsorbent material have not yet been developed to study all the main opium alkaloids present in poppy seeds.

The aim of this work was to develop and validate a rapid, easy and efficient method to determine the six main opium alkaloids, morphine, codeine, thebaine, papaverine, noscapine and oripavine in poppy seeds by UHPLC-QqQ-MS/MS. For this purpose, a SLE-MSPE sample preparation protocol was optimised using mesostructured silica-coated magnetic nanoparticles as the adsorbent. The method was successfully applied for the quantification of the six opioids in eleven poppy seeds and three seed mixes available in national food supermarkets to evaluate the levels of these alkaloids in seeds destined for food consumption.

## 2. Materials and Methods

### 2.1. Reagents and Materials

Standards of morphine, codeine, thebaine and oripavine were received from Alcaliber S.A.U. (Madrid, Spain). Noscapine, papaverine and morphine-D3 (internal standard) were obtained from Sigma-Aldrich (Sigma-Aldrich, Zwijndrecht, The Netherlands). Individual stock standard solutions were prepared at 1000 µg/mL in methanol. The intermediate mixed standard solution was prepared at 10 µg/mL in methanol. The working standard solutions were prepared at 1 µg/mL by diluting the intermediate mixed standard in methanol/water 50/50 (*v/v*). All of these were stored in darkness at −20 °C.

Ferric chloride 6-hydrate (FeCl_3_ 6H_2_O) 99% and ferrous chloride 4-hydrate (FeCl_2_ 4H_2_O) 99% were purchased from Labkem (Barcelona, Spain) and Acros Organics (Geel, Belgium), respectively. Tetraethylorthosilicate (TEOS) 98%, hexadecyltrimethylammonium bromide (CTAB) 98%, chloro(dimethyl)octylsilane (C_8_) and chloro(dimethyl)octadecylsilane (C_18_) were purchased from Sigma-Aldrich. HPLC grade acetonitrile, methanol and formic acid were purchased from Sigma-Aldrich. Ammonia (32%, *w/w*), isopropanol, toluene, ethanol and diethyl ether were of synthesis grade and acquired from Scharlab (Barcelona, Spain). Ultrapure water (resistance 18.2 MΩ cm) was obtained from a Milli-Q water purification system (Millipore, Billerica, MA, USA). The Nd-Fe-B magnet (5 × 5 × 2 cm) with force 200 kg used in the MSPE procedure was obtained from Superimanes S.L. (Sevilla, Spain).

### 2.2. Sample Collection

At the end of 2020, 14 samples of edible seeds were purchased in Spain from some supermarkets and herbalists, out of which three are mixtures of different edible seeds (pumpkin, sunflower, sesame, gold flax, brown flax and poppy seeds). In addition, for comparative purposes, two wild samples, one of white opium poppy (*Papaver somniferum* L.) and the other of corn poppy (*Papaver rhoeas* L.) were collected in Madrid and Zaragoza, respectively. Detailed information on each of these samples can be found in Table S1.

### 2.3. Synthesis of Organic-Functionalised Magnetic Particles

**Figure 1:**
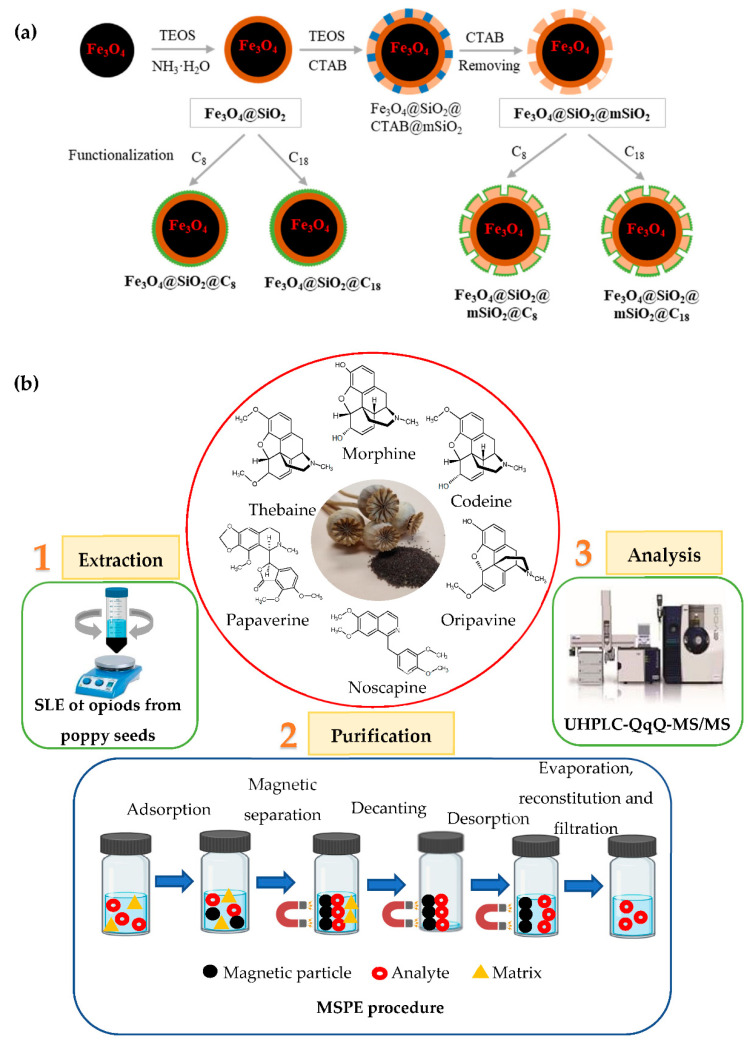
Schematic preparation process of the six types of magnetic particles synthesised (**a**). Diagram of proposed methodology to quantify opioids from poppy seeds: extraction (SLE), purification (MSPE) and analysis (UHPLC-QqQ-MS/MS) and chemical structures of the six most common opium alkaloids in contaminated poppy seeds (**b**).

Magnetic particles (Fe_3_O_4_) were coated in a first step with amorphous silica (Fe_3_O_4_@SiO_2_) and, after, with mesostructured silica (Fe_3_O_4_@SiO_2_@mSiO_2_). In addition, both materials were functionalised with organic groups (C_8_ or C_18_ ligand). The schematic preparation process of different magnetic particles is shown in Figure 1a.

#### 2.3.1. Preparation of Fe_3_O_4_ Particles

First, Fe_3_O_4_ particles were prepared using chemical co-precipitation according to the work of Zhang and Shi [28]. Briefly, 15 mmol FeCl_3_ 6H_2_O and 10 mmol FeCl_2_ 4H_2_O were dissolved in 80 mL of deoxygenated water while stirring at 300 rpm under nitrogen gas. The amount of 50 mL of ammonia solution (32%) was dropwise added into the clear yellow solution and it turned black. The reaction was maintained at 80 °C for 30 min. The black precipitates obtained (Fe_3_O_4_ particles) were then collected with the help of a strong magnet and washed repeatedly with deionised water until the pH of the washings became neutral and finally dried under vacuum at 60 °C for 24 h.

#### 2.3.2. Surface Modification of Fe_3_O_4_ Particles with Amorphous Silica (Fe_3_O_4_@SiO_2_)

The Fe_3_O_4_ particles were coated with amorphous silica (SiO_2_) according to the reported method of Zeng et al. with minor modifications [29]. Briefly, 1.5 g of Fe_3_O_4_ particles were dispersed in 60 mL of isopropanol/ultra-pure water (5/1, *v/v*) followed by ultrasonic dispersion for 20 min. Then, under continuous stirring, 15 mL of ammonia solution and 8 mL of TEOS were promptly added. After that, the mixture solution was stirred at room temperature for 24 h. Finally, the mixture was separated by an external magnetic field and the modified magnetic particles were washed with ultra-pure water, respectively, many times until the pH of the washing fluid was 7. Finally, the modified magnetic particles were dried under vacuum at 60 °C for 24 h.

#### 2.3.3. Surface Modification of Fe_3_O_4_ Particles with Mesostructurated Silica (Fe_3_O_4_@SiO_2_@mSiO_2_)

With the aim to obtain a material with a higher surface area that allows an increase in the functionalisation degree, Fe_3_O_4_@SiO_2_ particles were coated with a layer of mesostructured silica (mSiO_2_) according to the work of Deng et al., but with some modifications, as the amounts of the reagents were optimised [26]. Briefly, 3 g of Fe_3_O_4_@SiO_2_ was dispersed in a mixed solution containing 160 mL of ultra-pure water, 120 mL ethanol, 1.4 g CTAB and 12 mL ammonia solution. After the mixture was homogenised for 30 min, 6.4 mL of TEOS was dropwise added and stirred for 6 h. The product was collected with the help of a magnet and washed with ethanol and methanol several times. The Fe_3_O_4_@SiO_2_@CTAB@mSiO_2_ particles were collected with the aid of a magnet and were calcined to remove the CTAB using a heating program from room temperature to 550 °C at 1 °C/min and holding the temperature for 4 h.

#### 2.3.4. Organic Functionalisation of Fe_3_O_4_@SiO_2_ and Fe_3_O_4_@SiO_2_@mSiO_2_ Particles with C_8_ or C_18_ Groups

Both materials were functionalised with two different organic groups to compare. To carry this out, 0.5 g particles and 0.5 g C_8_ or C_18_ were added to 30 mL of anhydrous toluene under a nitrogen atmosphere. The mixture was stirred at 80 °C for 24 h. After the reaction, the particles were separated by an external magnetic field and were washed with toluene, ethanol and diethyl ether. Finally, the organic-functionalised magnetic amorphous and mesostructured particles were dried under vacuum at 60 °C for 24 h.

### 2.4. Characterization of Organic-Functionalised Magnetic Particles

Scanning electron microscopy (SEM) was recorded on Nova NanoSEM 230 FEI with an energy-dispersive spectrometry system (EDS). Previously, samples were treated with a sputtering method by dispersing the material in ethanol and coating the sample with a film thickness of 7 nm of gold. The samples were also characterised using XRD to evaluate the structure of the materials. Wide and low angle powder X-ray diffraction (XRD) patterns of the silicas were performed to determine if the material showed the typical spectrum of magnetite, which indicated that the magnetic core had not been disturb by the surface modification and if the material had a long mesoscopic ordered structure, respectively. XRD patterns were obtained on a Philips Diffractometer model PW3040/00 X’Pert MPD/MRD at 45 kV and 40 mA, using Cu Kα radiation (λ = 1.5418 Å). The samples were treated in power and placed in a sample holder. The sample and detector are rotated and the XRD patterns are collected from 0 to 10° in the low angle and between 20 to 70° in the wide angle. Iron and Silica wt% determination was carried out by X-ray fluorescence (XRF) using an X-ray fluorescence spectrophotometer Phillips MagiX with an X-ray source of 1 kW and a rhodium anode in a helium atmosphere. This quantification method can analyse from 0.0001% to 100% of Fe and Si. Infrared spectra were carried out on a Thermo Nicolet 380 Fourier-Transform Infrared (FT-IR) spectrophotometer in the region 4000–600 cm^−1^ using the ATR (Attenuated Total Reflection) technique to quantify the functional groups in the magnetic particles and to check the complete removal of the CTAB. For this, a small amount of sample (around 1 mg) previously vacuum-dried was used. The measures were performed at room temperature to avoid the signal of the physisorbed water and in the transmittance mode with 64 scans per spectrum at a resolution of 4 cm^−1^. Nitrogen gas adsorption–desorption isotherms were obtained using a Micromeritics ASAP 2020 analyser. These isotherms were measured at −196 °C over the interval of relative pressures (P/P_o_) from 10^−4^ to 0.994. Before measurements, the samples were degassed in a vacuum at 80 °C for 10 h in the port of degasification of the instrument. These temperatures were chosen to avoid any degradation of the organics groups and to remove adsorbed species, solvents and water. The Brunauer–Emmett–Teller (BET) method was utilised to calculate the specific surface areas (S_BET_). By using the Barrett–Joyner–Halenda (BJH) model, the pore volumes and pore size distributions were derived from the desorption branches of isotherms and the total pore volumes (V_t_) estimated from the desorbed amount at a relative pressure P/P_o_ of 0.97. In addition, this characterisation also allowed us to compare the surface areas and to optimise the amounts of TEOS and CTAB to be added at the mesostructured silica coating step. Finally, elemental analysis (% H, % C and % N) was performed using a microanalyser Flash 2000 Thermo Fisher Scientific Inc. (Hampton, NH, USA) to determine the degree of functionalisation.

### 2.5. Optimisation of UHPLC-QqQ-MS/MS Analysis

The determination of opium alkaloids was achieved with a UHPLC system (Advance Elute, Bruker, Billerica, MA, USA) equipped with a PAL RSI Autosampler (containing a loop of 100 μL) coupled to a triple quadrupole tandem mass spectrometer detector (EVOQTM Elite, Bruker) with an electrospray ion source (ESI). Chromatographic separation was performed on an Intensity Solo 2 C_18_ column (100 mm × 2.1 mm, Bruker, Billerica, MA, USA) at 30 °C. The injection volume was 10 μL (partial injection) and the flow rate was kept constant in the mobile phase at 0.4 mL/min during the analysis. A gradient elution was used by combining mobile phase A of water with mobile phase B of acetonitrile, both containing 0.1% formic acid. The linear gradient began in 95% A, in minute 3.5 changed to 30% A, in minute 3.7 returned to 95% B and then it was maintained in isocratic until minute 5.

The detection of each analyte was determined by direct infusion of a standard solution in methanol of 1 µL/mL of each opium alkaloid using a syringe pump at a flow rate of 20.0 µL/min. The mass spectrometer was operated in positive electrospray ion source mode (ESI+) with multiple reaction monitoring (MRM) using N_2_ as drying gas (350 °C and 40 psi); a cone temperature and gas pressure of 300 °C and 20 psi, respectively; Ar as nebuliser (60 psi); and collision gas (2 mTorr) and ion spray voltage of 4200 V; collected delay at 0.6 min with a detector voltage at 1.65 V. Compounds were monitored with the transitions shown in Table S2.

### 2.6. Optimisation of Sample Preparation

For the optimisation of the sample preparation, firstly, the SLE parameters and, secondly, the MSPE purification parameters were optimised. The diagram for each of these steps is shown in Figure 1b.

#### 2.6.1. Optimisation of SLE of Opioids from Poppy Seeds

First, the conditions for the SLE of opioids from poppy seeds were examined. The parameters that were optimised were the following: the mass of initial seeds (1, 2.5, 5 and 10 g); the type of solvent (acetonitrile/water/formic acid 80/19/1, *v/v/v*, methanol, methanol/water 50/50, *v/v* and methanol 0.1% acetic acid); the agitation type (ultrasound and magnetic stirring); the volume of solvent (10, 20 and 30 mL); the extraction time (10, 20, 30 min and 1 h); the number of successive extractions (up to 6); and the pH of the solvent (3, 5, 6.8 and 10).

#### 2.6.2. Discontinuous Adsorption Studies to Select the Best Magnetic Material for MSPE Procedure

Secondly, discontinuous adsorption studies were performed with all the previously synthesised magnetic materials to evaluate their adsorption capacity and to choose the most suitable one for the subsequent MSPE process. For this purpose, 100 mg of each of the six synthesised materials was added to 2 mL of different types of solvents (methanol/water 50/50, *v/v*, acetonitrile/water/formic acid, 80/19/1, *v/v/v* and methanol 0.1% acetic acid) containing 1 µg/mL of each of the six analytes. Finally, the supernatants of each of the different solvent type were analysed by UHPLC-QqQ-MS/MS after a determined adsorption time (0, 1, 5, 10 and 20 min). All the studies were made in duplicate and the adsorption percentages of each of the materials on the different solvents were calculated and compared to determine which material and solvent were the best.

#### 2.6.3. Adsorption Kinetic and Isotherm Experiments with Fe_3_O_4_@SiO_2_@mSiO_2_ Material

The adsorption kinetics and isotherms were investigated with the material selected for the MSPE procedure.

For adsorption kinetics, 100 mg of the material was added to a solution containing each of the six analytes (2 mL, 1 µg/mL). The mixtures were shaken at different times (1–20 min) and the supernatants in equilibrium were analysed by UHPLC-QqQ-MS/MS. The adsorption capacity was calculated by Equation (S1) in Table S3. The adsorption kinetics were determined by Langergren’s pseudo-first-order [30], pseudo-second-order [31] and intra-particle diffusion kinetic models [32] (Table S3).

For adsorption isotherm, a series of 2.0 mL solutions of different concentrations of the six analytes (0.1–50 µg/mL) were added to 100 mg of the material under optimum time. The isotherms of the six opium alkaloids adsorption on the magnetic particles were analysed using the commonly used Langmuir [33] and Freundlich [34] models (Table S3).

#### 2.6.4. Optimisation of MSPE Conditions with Fe_3_O_4_@SiO_2_@mSiO_2_ Material

Conditions of the MSPE procedure were optimised. For this purpose, 2 mL of 1 µg/mL of each of the six analytes was used. The parameters of the MSPE procedure that were optimised were the following: the amount of adsorbent (between 25 and 100 mg); the adsorption time (from 1 to 20 min); the pH of the initial solution (ranging from 3 to 10); the desorption eluent (diethyl ether, dichloromethane, chloroform, isopropanol, acetonitrile, methanol, water, ethyl acetate and ethanol) with different percentages of acid (formic acid) or base (ammonia) between 0.1 and 20% or mixtures of some of these solvents at different proportions (50/50, 80/20 or 20/80, *v/v*); the volume of the desorption eluent (from 2 to 5 mL); and the number of successive desorptions (up to 5). All studies were carried out in triplicate. One factor at a time method was employed to obtain the optimal conditions of each parameter.

### 2.7. Optimised Sample Preparation Procedure

#### 2.7.1. Optimised SLE of Opioids from Poppy Seeds

In the SLE of opioids from poppy seeds, 2.5 g of seeds were extracted with 30 mL of methanol/water, 50/50 (*v/v*). The mixture was vortexed for 30 s and for 30 min it was stirred magnetically. Later the supernatant was recovered and a second extraction was carried out in the same conditions. Finally, the two supernatants were put together and 2 mL were taken for purification through the MSPE.

#### 2.7.2. Optimised MSPE Procedure

The steps that were carried out in the MSPE procedure are shown in Figure 1b. In the first place, 50 mg of Fe_3_O_4_@SiO_2_@mSiO_2_ were added with the extraction supernatant and then the mixture was treated with 1 min ultrasonication until the analytes arrived at an adsorption equilibrium with the adsorbent material. Then, with the help of a magnet, the solution was decanted and the analytes were eluted from the magnetic particles with 2 mL of diethyl ether/methanol 80/20, *v/v* by ultrasound for 1 min. Later the supernatant was recovered and a second desorption was carried out in the same conditions. Finally, 2 mL of these supernatants with the analytes was vacuum evaporated, 50 µL of a 1 µg/mL solution of morphine-D3 was added, reconstituted in 1 mL and then filtered and analysed by HPLC-QqQ-MS/MS.

### 2.8. Instrumental and Method Validation

The present methodology was validated in terms of linearity, method and instrumental detection (LOD, MDL) and quantification (LOQ, MQL) limits, matrix effects, accuracy, precision and selectivity. For more details, see section S1.1 in Supplementary Materials. Since there is no official regulation for validation methodologies for quantified opium alkaloids in poppy seeds, the validation was conducted according to the criteria established in SANTE/11813/2017 document for the analytical quality control of pesticide residues in food and feed [35] and EC No 401/2006 [36] establishing the methods of sampling and analysis for the official control of the levels of mycotoxins in foodstuffs.

For the validation process, the commercial poppy seed sample PS05 (Table S1) was used since it had low levels of alkaloids. In addition, a double wash with water at 100 °C for 30 min was applied. Signals of the analytes obtained were subtracted when required.

## 3. Results and Discussion

### 3.1. Characterisation of Magnetic Materials Synthetised

#### 3.1.1. SEM

The morphology of the synthesised particles can be observed from the SEM images in Figure S1. The particles have a spherical morphology and have resulted in a considerable growth in size after the surface modification. The initial Fe_3_O_4_ particles have a size of approximately 20 nm and the modified particles with amorphous silica (Fe_3_O_4_@SiO_2_) have a size in the range of 0.55–0.91 µm and with mesostructured silica (Fe_3_O_4_@SiO_2_@mSiO_2_) of 1.00–1.36 µm. This demonstrated the successful modifications of material as there is a growth in size with the first coating and another growth with the second coating.

#### 3.1.2. XRD

The XRD patterns of the synthesised magnetic particles are shown in Figure S2. The low-angle XRD pattern reveals the Miller index (100) characteristic of materials with mesoscopic order. In addition, there are several relatively strong diffraction peaks in the 2θ region of 20–70°, which are similar to those of the Fe_3_O_4_ particles reported by other groups [26,37,38], with the six discernible diffraction peaks 220, 311, 400, 422, 511 and 440 that correspond with the Miller index diffraction peaks that appears in the database of magnetite in JCPDS (JCPDS card: 19-629) file. This finding proved that all the silica-coated magnetic particles were composed of Fe_3_O_4_ core. Moreover, the peak positions of the XRD patterns between different materials remain unchanged, which indicates that the magnetic core had not been disturb by the surface modification. This is also observed during the MSPE procedure. Although the separation rate was gradually reduced with the surface modification of Fe_3_O_4_, the magnetism of the functionalised particles is still strong enough that magnetic decantation with the external magnet can be performed quickly and without sample loss.

#### 3.1.3. XRF

X-ray fluorescence measurement was made to determine the concentration (%) of iron and silica in all the materials synthesised. The percentage of iron decreased from 57.8 in Fe_3_O_4_ to 31–34% in the functionalised particles covered with amorphous silica (with 15–14% Si) and to 13–20% (with 20–19% Si) in the mesostructured silica shell functionalised particles. Nevertheless, the amount of iron in the materials after functionalisation is still large enough to confer high magnetism and to ensure a quick and easy separation of the microparticles from the solution in the MSPE procedure with the use of a strong magnet.

#### 3.1.4. FT-IR

An important aspect to consider before the functionalisation of the particles with the C_8_ or C_18_ ligands is the successful removal of the CTAB. To perform this, the amount of CTAB present in the materials was monitored after the removal treatment by using FT-IR spectroscopy before the functionalisation process by checking the presence of the bands of the -CH_2_ and -CH_3_ groups from CTAB at 2855 cm^−1^ and 2925 cm^−1^, respectively. The first treatment was performed with a Soxhlet acetone extraction, as in most studies [22,23,36,37]. However, the intensity of these bands was remarkable (Figure S3a, spectrum 2), which showed that this treatment was not effective in eliminating the surfactant. For this reason, in this work, we use a calcination treatment as an effective method for the complete elimination of rest of CTAB remaining inside the porous structure of the materials. This was confirmed by the absence of the C-H bands, as can be observed in Figure S3a, spectrum 1.

In addition, the materials synthesised in this study were characterised by ATR-FT-IR. The results of each magnetic material are shown in Figure S3b. The absorption peak that can be seen starting around 600 cm^−1^ in all the spectra was attributed to the Fe-O-Fe vibration. This band confirms the existence of a magnetic core of Fe_3_O_4_ in all the particles. The successful surface modification with silica of the magnetic particles can be confirmed with the appearance of the Si-O-Si band at 1090 cm^−1^ in all the modified materials. After functionalisation, the organic groups of C_8_ and C_18_ showed two new peaks appearing at 2855 cm^−1^ and 2925 cm^−1^ due to the C-H stretching bands of the -CH_2_- and -CH_3_ groups. The peak intensity of these bands in the materials functionalised with C_18_ is higher than that functionalised with C_8_ because of the larger number of -CH_2_- and -CH_3_ groups in the C_18_ ligand (Figure S3b, spectrums 4 and 5, 6 and 7). Moreover, the intensity of this band is higher in the particles modified with mesostructured silica than with amorphous silica (spectrum 6 compared to 4 and 7 compared to 5), which can be attributed to a higher functionalisation degree, which is in accordance with the results obtained in the elemental analysis (Table 1).

#### 3.1.5. BET

BET characterisation was used to optimise the amount of TEOS and CTAB for coating the magnetic core with a mesostructured silica layer. When coating the Fe_3_O_4_ particles with the amounts of TEOS and CTAB used in the literature [26], a considerable loss of magnetism of the particles was observed and, therefore, much of the material was lost in the washing stage of the material by magnetic decantation, which resulted in a very low yield of the synthesis. In the washes, it was visually observed that different fractions of particles were formed, some were more coated with mesostructured silica than others and therefore with different levels of magnetism. As shown in Figure S4, the first fraction was darker brown and the second and third (less magnetic) fractions were considerably more whitish due to an excess of the silica layer. In order to check this, BET surface area measurements were performed on the first three fractions, showing the following surface area results: 380 m^2^/g for the first wash, 835 m^2^/g for the second wash and 790 m^2^/g for the third wash. This showed that TEOS and CTAB were added in excess, resulting in a lower yield and a non-homogeneous coating; therefore, in order to avoid this issue, the contents of these two reagents were reduced. The new amounts were compared with the original amount [26], with half and with a quarter of each of the two reagents, and the surface areas were compared. It was found that the surface area did not change significantly (219, 205 and 314 m^2^/g, respectively) and that decreasing the amount of reagent resulted in a higher yield since fewer particles were lost by magnetic decantation. Thus, syntheses of Fe_3_O_4_@SiO_2_@mSiO_2_ were carried out with a quarter of the amounts of TEOS and CTAB used in the literature [26].

In addition, Fe_3_O_4_@SiO_2_ and Fe_3_O_4_@SiO_2_@mSiO_2_ were characterised before and after functionalisation. The N_2_ adsorption–desorption isotherms of the materials synthesised in this work are shown in Figure S5a and c. For all materials, its adsorption isotherm can be assigned as Type IV, according to the IUPAC classification [39], which is characteristic of mesoporous material. The isotherms show an initial part that is characteristic of monolayer adsorption and a significant increase in the amount adsorbed at intermediate relative pressures characteristic of a multilayer filling mechanism. In the case of Fe_3_O_4_@SiO_2_ and their functionalised derivatives, they presented an H3 hysteresis loop with almost parallel branches confined to relative pressures between 0.8 and 1. This hysteresis loop is characteristic of uniform pores with a slit-like structure. Results obtained a BET specific surface area (S_BET_) of 147 m^2^/g and a pore volume of 0.18 cm^3^/g for the non-functionalised and 26–24 m^2^/g and 0.09–0.10 cm^3^/g for the functionalised with C_8_ and C_18_, respectively, which demonstrates the correct functionalisation of the materials (Table 1). As shown in Figure S5b, materials with amorphous silica have a disorganised pore distribution (width of the peak at half of the height equals to 100 Å) because no template was used. Non-functionalised material presents most of its mesopores centred at 125 Å and a small group of mesopores can also be observed at 35 Å. After the C_8_ and C_18_ functionalisation, the small pores at 35 Å disappear and only the big pore at 125 Å is shown (Figure S5b and Table 1). The reason for this is that a decrease in the pore volume and pore diameter takes place, which is indicative of the correct anchorage of both ligands into the pores of the materials.

On the other hand, the Fe_3_O_4_@SiO_2_@mSiO_2_ and their functionalised derivatives also reflected Type IV adsorption isotherm, but in this case with an H4 hysteresis loop. Results obtained a S_BET_ of 355 m^2^/g and a pore volume of 0.23 cm^3^/g for the non-functionalised and 191–14 m^2^/g and 0.14–0.04 cm^3^/g for the functionalised with C_8_ and C_18_, respectively. This shows the correct functionalisation of the material and that the C_8_ ligand provides a greater surface area due to its smaller chain. The pore size distribution is close to the micropore range, unlike materials with amorphous silica. As can be observed in the pore size distribution (Figure S5d), these materials present most of their pores centred around 40 Å.

#### 3.1.6. Elemental Analysis

The percentage of C in each functionalised material, calculated by elemental analysis, showed different levels of functionalisation depending on the silica and organic group type, as shown in Table 1. Consequently, the material with the highest level of functionalisation was Fe_3_O_4_@SiO_2_@mSiO_2_@C_8_ with 0.869 mmol/g, followed by Fe_3_O_4_@SiO_2_@mSiO_2_@C_18_ with 0.471 mmol/g, Fe_3_O_4_@SiO_2_@C_8_ with 0.286 mmol/g and, finally, Fe_3_O_4_@SiO_2_@C_18_ with 0.134 mmol/g, which is coherent seeing the S_BET_. By observing these results, it can be confirmed that the use of mesostructured silica permits a higher level of functionalisation than amorphous silica. Concerning the ligand, the one that provides the higher levels is C_8_. This may be because it is less voluminous due to its shorter chain and has greater accessibility through the pores of the material, allowing for a higher level of functionalisation.

### 3.2. Optimisation of UHPLC-QqQ-MS/MS Analysis

Triple quadrupole mass spectrometric parameters were optimised for analytes in positive ionization mode by direct infusion of a standard solution in methanol of 1 µg/mL of each opium alkaloid using a syringe pump at a flow rate of 20.0 µL/min. The molecular ion of each compound was detected with a Q_1_ resolution of 0.7 at a scan time of 500 ms and to obtain the maximum intensity of the fragment ions of each analyte while the collision energy was optimised. For chromatographic separation, two mobile phases were tested by using acetonitrile or MeOH as eluent B and, in both cases, water as eluent A, with 0.1% formic acid for both. The results were better with acetonitrile than methanol because of their best peak intensity and separation. Successive gradients were tested and, finally, the separation of the six analytes was achieved in only 5 min with the following gradient: 5–70% B (0–3.5 min), 70–5% (3.5–3.7 min) and 5% (3.7–5 min). Table S2 lists the parent ion, daughter ion and collision energy (eV) optimal for MRM detection mode and the retention time of the opium alkaloids.

### 3.3. Optimisation of Sample Preparation

#### 3.3.1. Optimisation of SLE of Opioids from Poppy Seeds

Many protocols in the literature have been examined for the extraction of opioids from poppy seeds. Some of them have used a large amount of sample and organic solvents, for example, a double extraction with 100 mL of acetonitrile/water/acid formic 80/19/1 (*v/v/v*) to extract 10 g poppy seeds [1]. In order to minimise the costs and negative impact on the environment, an attempt was made to reduce the amount of sample to be used and, with it, the amount of organic solvents. For this reason, the first SLE experiment was carried out in triplicate with 1, 2.5, 5 and 10 g of sample with 10 mL of acetonitrile/water/acid formic 80/19/1 (*v/v/v*) and for four different extraction times (2.5, 5, 10 and 20 min). As a result, very high RSD values, especially with 1 g sample (mean values for all analytes were 82, 66, 53 and 71% for each time, respectively), were observed. However, with 2.5 g of sample, the RSD was lowered (between 16 and 26%) but was not improved with 5 and 10 g of sample. This dispersion in the results could be due to the variability in the opioid content of seeds from the same batch, as the contamination of each seed with plant latex could not be the same as it depends on many different factors (mainly genetic factors and environmental conditions) so that their contamination is finally very heterogeneous. Therefore, it was decided that it was sufficient to use a 2.5 g sample in order to minimise the use of environmentally harmful organic solvents.

Concerning the extraction solvent in the literature, many authors use methanol to extract opioids from poppy seeds [9,17,40,41,42]. Others use acidified methanol [6,43] or a mixture of acetonitrile/water/formic acid 80/19/1(*v/v/v*) [1]. For this reason, in the present study, these three types of solvents were evaluated in two agitation modes, ultrasound (US) and magnetic stirring with 2.5 g of poppy seeds and 10 mL of solvent during 10 min. In addition, the solvent methanol/water 50/50 (*v/v*) was added because it was the medium where the standards were dissolved and conserved in a stable form. As shown in Figure S6, not much difference was shown between acetonitrile/water/formic acid 80/19/1 (*v/v/v*) and methanol/water 50/50 (*v/v*) (although acetonitrile was better for morphine and noscapine). Thus, the solvent finally used for this purpose will depend on the medium that best favours the subsequent adsorption step in the MSPE procedure to avoid the evaporation process and to use less organic solvent.

Successive extractions were then performed to find out how many extractions were needed to complete the extraction. Six successive extractions were carried out with 20 mL of methanol/water 50/50 (*v/v*) for 10 min under magnetic stirring and complete extraction was not obtained. In the first extraction, most of the opioids present in the seeds were extracted and, from the third extraction, the values obtained were very low and constant and, thus, it was not considered from the third extraction onwards. Furthermore, the same study was carried out with acetonitrile/water/formic acid, 80/19/1 (*v/v/v*) and similar results were obtained. Therefore, a double extraction with methanol/water 50/50 (*v/v*) was proposed.

Afterwards, the solvent volume and the extraction time were optimised. For this purpose, different combinations of double extractions were made with methanol/water 50/50 (*v/v*) in magnetic stirring where the volumes tested were 10, 20 and 30 mL and times 10, 20, 30 min and 1 h. As shown in Figure S7, as the solvent volume increased and the amount of opioids extracted increased considerably. In the same manner, the more time was used, the more was extracted, except from 30 min to 1 h where there was no increase in extraction. Furthermore, once the extraction volume and time were optimised, an additional third extraction was performed and only one residual area was observed; therefore, a decision was made to perform only two extractions of 30 mL during 30 min.

Finally, the pH value of the extraction solvent (pH 3, 5, 6.8 and 10) was studied. Thus, double extraction of 30 mL methanol/water 50/50 (*v/v*) for 30 min in magnetic stirring with different pH value: 3, 5, 6.8 (non-modified) and 10 was carried out. In order to obtain the acidified pH, formic acid was used and, for the basic pH, ammonia. As can be observed in Figure S8, with the unmodified solvent (pH 6.8) higher extractions were obtained for all the analytes.

#### 3.3.2. Selecting the Best Material for the MSPE Procedure after Making Discontinuous Adsorption Study with the Synthesised Magnetic Materials

**Figure 2:**
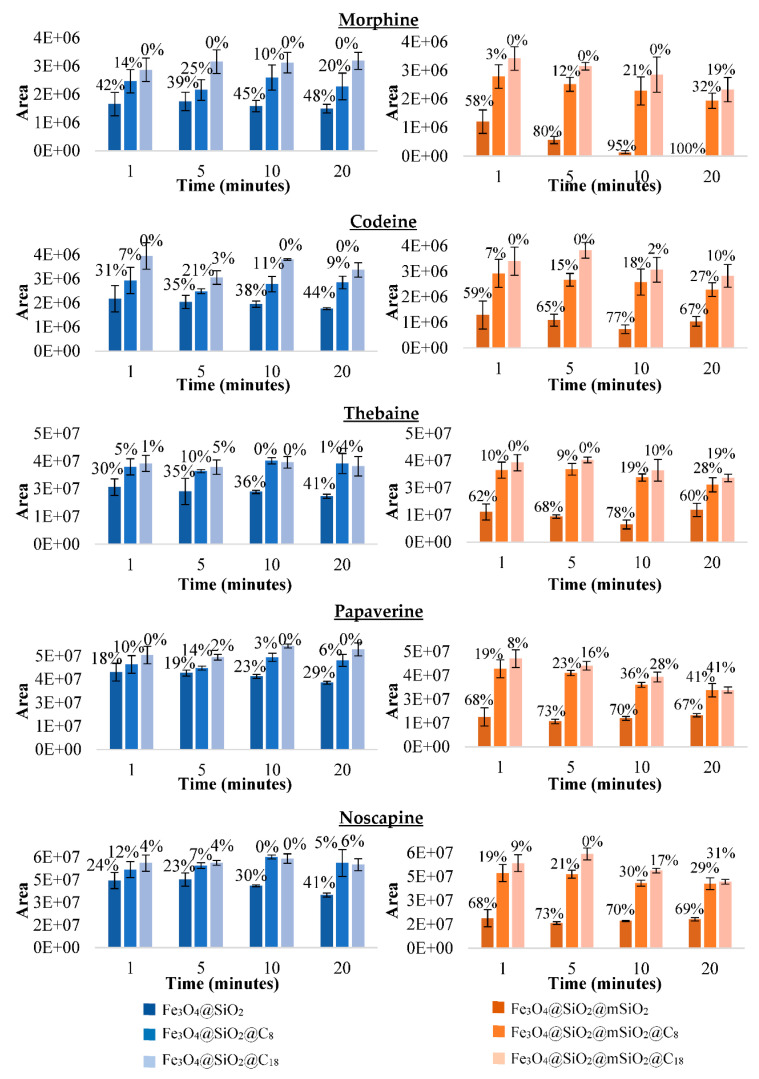
Areas of the supernatant with their respective percentages of adsorption (%) at different times with each of the three amorphous silica materials (**blue colour**) and mesostructured silica magnetic materials (**orange colour**) with methanol/water 50/50 (*v/v*) as the adsorption solvent.

In order to select the best magnetic material to conduct the MSPE procedure, a discontinuous adsorption study was carried out with 2 mL of methanol/water 50/50 (*v/v*), with 1 µg/mL of each of the six analytes and 100 mg of each of the material synthetised. This adsorption medium was selected because it was the medium where the working standard solutions were prepared and were, therefore, soluble and stable and 100 mg of material was used because it was the maximum quantity of the material expected to carry out the MSPE procedure. As can be seen in Figure 2, the result was that the three materials synthesised coated with mesostructured silica showed lower area values of the supernatant as the adsorption time increases and, with it, higher percentages of adsorption than amorphous silica materials. This agrees with the characterisation results since the mesostructured silica-coated material had a larger surface area than amorphous silica-coated material (355 vs. 147 m^2^/g). In addition, more adsorption was obtained with non-functionalised materials than functionalised materials with C_8_ and C_18_ group.

**Figure 3:**
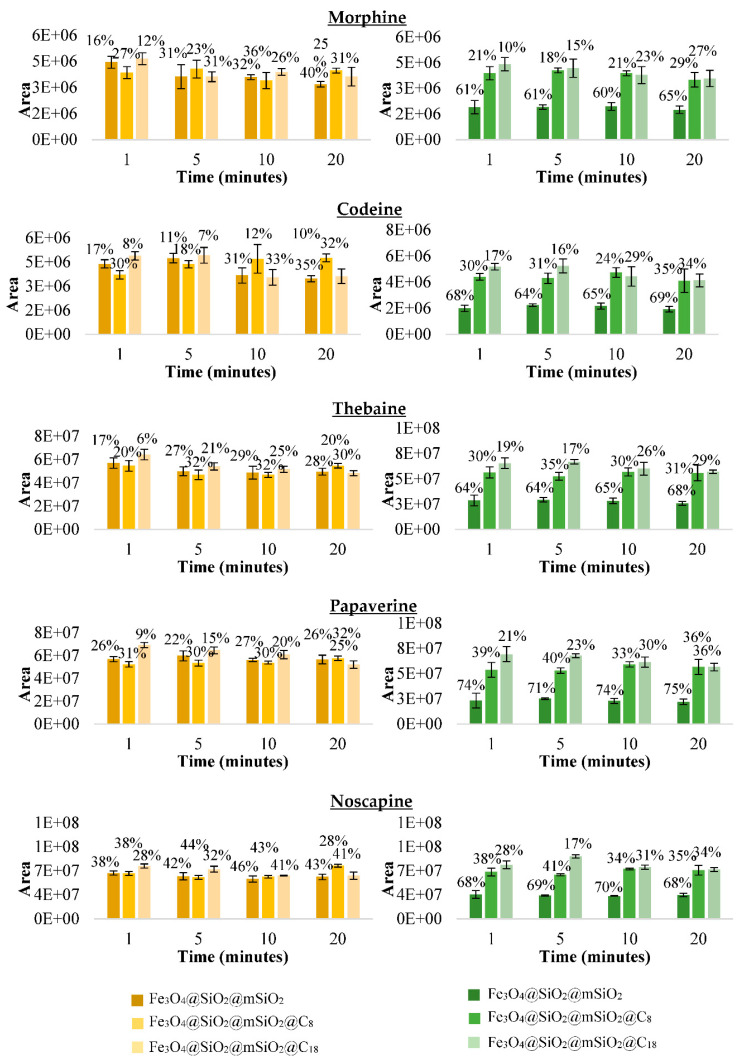
Areas of the supernatant with their respective percentages of adsorption (%) at different times with each of the three mesostructured silica magnetic materials with different adsorption solvent: acetonitrile/water/formic acid 90/19/1 (*v/v/v*) (**yellow colour**) and methanol acidified with 0.1% acid acetic (**green colour**).

In order to confirm the higher adsorption of non-functionalised material, an additional experiment was carried out with the three mesostructured materials with different adsorption media. The solvents selected to carry out the adsorption step with the material were the two solvents that showed similar results in the extraction of the opioids from the seeds, acetonitrile/water/formic acid 80/19/1 (*v/v/v*) and methanol 0.1% acetic acid (Figure 3). Therefore, the results obtained previously with methanol/water 50/50 (*v/v*) were compared with the results obtained with acetonitrile/water/formic acid 80/19/1 (*v/v/v*) and with acidified methanol 0.1% acetic acid. By comparing Figure 2 (orange colour) and Figure 3, with all solvents, more adsorption (%) was observed with the non-functionalised material. This can be due to two possible reasons: When functionalising, the pore size has been reduced so much that the molecules do not penetrate and only a surface interaction takes place or the analytes do not interact effectively with the C_8_ and C_18_ groups. Therefore, to carry out the MSPE, the material Fe_3_O_4_@SiO_2_@mSiO_2_ was finally selected. In addition, the results were significantly better with methanol/water 50/50 (*v/v*) than with the other two solvents. Therefore, this solvent mixture was selected to carry out the adsorption step of the MSPE as for the extraction of opioids in the poppy seeds to avoid the evaporation step.

#### 3.3.3. Adsorption Kinetic and Isotherm Experiments of Fe_3_O_4_@SiO_2_@mSiO_2_ Material

As shown in Figure S9a, the adsorption kinetics of the six opioid alkaloids is fast. In the first minute, almost all the adsorption is obtained and, in the following minutes, it was similar. However, in morphine and oripavine, there is a considerable increase in adsorption at minute 20 with respect to minute 1 and so it was decided to take 20 min as the adsorption time for the rest of the experiments at the moment. The adsorption kinetics were determined by Langergren’s pseudo-first-order [30], pseudo-second-order [31] and intra-particle diffusion kinetic models [32], according to the Equations (S2)–(S4) in Table S3. The results were shown in Figure S10a and the important data of the three kinetic models were compiled in Table S4. The linear regression coefficients (R^2^) were more close to 1 in the pseudo-second-order model and their Q_e, cal_ (calculated result) was more similar to Q_e, exp_ (experiment result). For these reasons, the adsorption of the six opium alkaloids accorded mostly to the pseudo-second-order kinetics, indicating a chemical adsorption mechanism [37]. In addition, morphine and oripavine were the analytes with the highest intraparticular diffusion rate with K_p_ values of 1.97 and 1.53 mg/g min^2^, respectively, and showed R^2^ close to 1 (Table S4), unlike the rest of the analytes that did not show this trend.

The adsorption isotherms were analysed by Langmuir [33] and Freundlich [34] models, according to the Equations (S5) and (S6) in Table S3. As shown in Figure S9b, by increasing the concentration of opioids in the equilibrium, the adsorption capacity was increased. In morphine, the maximum adsorption capacity was determined but, in the other opioids, it was still possible to adsorb more. As shown in Figure S10b, the R^2^ obtained by the Langmuir model was more close to 1 than by the Freundlich model. Thus, the adsorption occurred on the uniform monolayer surface of Fe_3_O_4_@SiO_2_@mSiO_2_ [37]. Equations (S1), (S5) and (S6) allowed the calculation of Q_max_ and was 73.59, 102.04, 303.03, 303.03, 107.353 and 149.25 mg/g for morphine, codeine, thebaine, papaverine, noscapine and oripavine, respectively.

#### 3.3.4. Optimisation of MSPE Procedure with Fe_3_O_4_@SiO_2_@mSiO_2_ Material

##### Adsorption Conditions (Time and pH)

In order to determine the optimal adsorption time, different time ranges between 1 and 20 min were investigated with 100 mg Fe_3_O_4_@SiO_2_@mSiO_2_ material. It can be seen in the adsorption kinetics of six opioid alkaloids in Figure S9a that the amounts of alkaloids adsorbed (Q_t_) at 1 min are similar to those adsorbed at 20 min, except for morphine and oripavine which have slower adsorption kinetics and, at 20 min, their maximum adsorption takes place. After determining that these two compounds have a higher intraparticle diffusion than the rest of the analytes (Section 3.3.3), in addition to evaluating the adsorption time in the highest or lowest level of adsorption of the analytes on the material, the influence of the adsorption time on the complete MSPE process was studied. For this purpose, the recoveries obtained at 1 and 20 min were calculated. In order to calculate them, the areas obtained by performing the complete MSPE procedure on a sample at a known concentration were compared with the areas of a blank sample subjected to the same extraction and purification process and spiked with the expected concentration. Finally, the recoveries of morphine and oripavine obtained after performing the complete MSPE procedure were lower at 20 min adsorption than at 1 min adsorption, approximately 20% versus 50%, respectively. The possible reason for this may be that, with 20 min adsorption, a higher percentage of adsorption is obtained, but it penetrates so much into the pores (following the intra-particle model studied in Section 3.3.3) that later, in the desorption step, it cannot be desorbed. For this reason, the adsorption time of 1 min was selected for the MSPE procedure.

The influence of the pH of the sample solution on the efficiency of the MSPE process was investigated. For this purpose, 1 µg/mL of each of six opioids was in contact with 100 mg Fe_3_O_4_@SiO_2_@mSiO_2_ material in methanol/water 50/50 (*v/v*), with different pH values between 3.0 and 10.0 were studied for 1 min adsorption. Such as shown in Figure S11, there were no major differences between the values, especially in morphine and oripavine, which were very similar. In the other analytes, it was observed that the pH 6.8 of the methanol/water 50/50 (*v/v*) without modification was the one that showed increased adsorbed area and with lower RSD, since the 7.6 showed more variation. For codeine, pH 6.8 and 10 showed no differences and, for thebaine, pH 10 facilitated the adsorption but, for papaverine and noscapine, it decreased and finally pH 6.8 was selected as the best.

##### Desorption Conditions (Solvent Type, Time, pH and Number of Consecutive Desorptions)

The type of the desorption solvent selected is a critical parameter to obtain the highest possible desorption of the analytes and thus a higher recovery value (%). First, the four solvents used in the SLE of poppy seeds at different desorption times (1, 5, 10 and 20 min) were used with 20 min of adsorption time. As shown in Table S5, the recoveries obtained were low for morphine and oripavine. Therefore, it was determined that none of these solvents were effective for desorption. Consequently, eight solvents were studied (diethyl ether, dichloromethane, chloroform, isopropanol, acetonitrile, methanol, water and ethyl acetate) at 20 min desorption. All of them were used at three pH values, acidic with formic acid 1%, without modification and basic with ammonia 1%. With the first two conditions, it was not possible to desorb the analytes from the material obtaining low recoveries, for morphine and oripavine (around 1%) and for the rest of the analytes less than 50%. While the basic medium facilitated the desorption of the six opioids, the recoveries obtained were still low. Therefore, it was decided to increase the basicity of the medium by adding ammonia 10%. As can be observed in Table S6, by increasing the ammonia content, the recoveries improved moderately. However, the recoveries were still low and so different mixtures were performed with the solvents that produced the best results, which included diethyl ether with methanol or acetonitrile and dichloromethane with methanol or acetonitrile at different proportions (50/50, 80/20 and 20/80, *v/v*) and for different times (20, 40 and 60 min). As shown in Table S7, the best combination was diethyl ether/methanol, 80/20 (*v/v*) as all analytes were around 100% recovery, except for morphine and oripavine, for which the maximum recovery was 37%. The reason why desorption of these two analytes was so difficult might be because they showed high intraparticle diffusion (Section 3.3.3). Finally, the adsorption and desorption times were reduced and the study was carried out with 1 min of adsoption and with 1 min for up to five consecutive desorptions with diethyl ether/methanol 80/20 (*v/v*) with 10% ammonia. As can be observed in Table S8, higher recoveries were obtained with short times in adsorption and desorption steps, between 76 and 109% for all analytes, except for morphine and oripavine which reflected recoveries near 50%. In addition, the increase the desorption solvent volume to double was also studied from 2 mL to 4 mL. However, no better results were obtained. Therefore, the best option was to make triple desorption with 2 mL for 1 min.

##### Amount of Fe_3_O_4_@SiO_2_@mSiO_2_ Material

**Figure 4:**
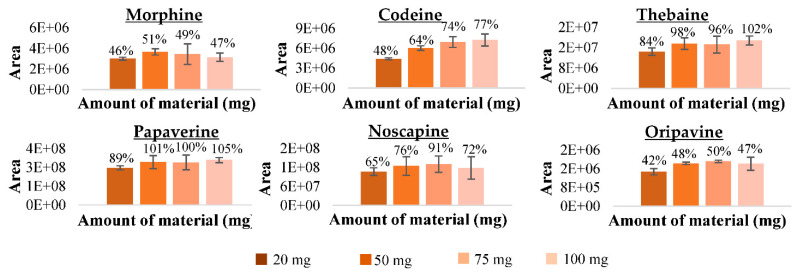
Effect of the different amounts of material in the optimised MSPE procedure with Fe_3_O_4_@SiO_2_@mSiO_2_ material.

In order to make the MSPE procedure effective and to obtain good results, different amounts of magnetic particles between 25 and 100 mg were studied. Figure 4 shows the areas of desorbed opium alkaloids achieved with the different amounts of adsorbent material with their respective recovery values (%). The amount selected to perform the MSPE procedure was 50 mg of magnetic particles because with this amount 51, 64, 98, 101, 76 and 48% recoveries for morphine, codeine, thebaine, papaverine, noscapine and oripavine, respectively, were achieved. Increasing the amount of adsorbent material above this did not significantly improve the results.

### 3.4. Instrumental and Method Validation

The instrumental validation parameters are displayed in Table S9. First, the linear range of each of the six analytes dissolved in pure solvent was evaluated. Instrumental limits of detection and quantification were calculated by analysing dilutions with decreasing concentration until the S/N ratio was approximately 10. The LOQ and LOD were estimated as 10 and 3 times the S/N ratio, respectively, obtaining the LOQ and LOD for morphine, codeine and oripavine around 8 × 10^−2^ and 2 × 10^−2^ µg/L, respectively, and for thebaine, papaverine and noscapine around 5 × 10^−3^ and 1 × 10^−3^ µg/L, respectively (Table S9). The linear range started at 0.1 µg/L for morphine, codeine and oripavine; or at 0.01 µg/L for thebaine, papaverine and noscapine up to 5000 µg/L.

The method validation parameters are displayed in Table 2. The MDL and MQL for morphine, codeine, papaverine and noscapine were 0.07 and 0.24 µg/kg; and for thebaine were 0.72 and 2.4 µg/kg; and for oripavine were 72.07 and 240 µg/kg, respectively. The linear range started at 0.01 µg/L for morphine, codeine, papaverine and noscapine; at 0.1 µg/L for thebaine; or at 10 µg/L for oripavine up to 5000 µg/L. Calibration lines were obtained with an adequate R^2^ (0.999). In addition, the Cm was calculated, which was always ≥92%, successfully accomplishing the criteria established on the guidelines [35].

In order to evaluate the ME, the slopes of each of the calibration lines were compared. As shown in Table 2, the ME (%) obtained when comparing the slopes of the purified matrix versus the slope of the solvent ranged from 80–109% for codeine, thebaine and papaverine; thus, these analytes are not affected by any matrix effect that may remain after purification (according to the criteria established in SANTE/11813/2017 [35]). For morphine, noscapine and oripavine, values slightly less than 80% were obtained, indicating that there was signal suppression. Therefore, to quantify the target analytes in the real samples, matrix-adjusted calibration curves had to be used to compensate for the errors associated with these matrix effects. In addition, a matrix calibration curve without MSPE purification was also analysed and, in this case, the ME (%) calculated was in almost all analytes more than 120%, especially for thebaine and oripavine, which were 294 and 258%, respectively. For this reason, a considerable improvement was observed after MSPE purification, which allowed the matrix interferences to be reduced.

Accuracy was expressed as the average recovery obtained from six samples (*n* = 6) spiked with the analytes at a known concentration and subjected to the proposed extraction-purification procedure. Accuracy was evaluated at two concentration levels: low (0.1 mg/kg) and high (1 mg/kg). Recovery values were calculated by comparing the value of the samples with the value of the simulated samples (samples subjected to the same extraction and purification process but spiked at the same concentration level prior to chromatographic analysis). As shown in Table 2, the recovery values obtained were in the range 70–116%, thus favorable according with the guidelines [35] However, for morphine and oripavine, the recoveries obtained were around 50%, which may be due to the fact that they were the smallest analytes and displayed higher intraparticle diffusion and were, therefore, more difficult to desorb, resulting in lower recoveries. As the molecules were smaller in size, the adsorbent material adsorbed the analytes but they were not completely desorbed as they remained in the internal and smaller pores of the material due to the fact that they are the compounds with the highest intraparticle coefficient (see Section 3.3.3). In order to check that the recovery value of morphine was associated with the nature of the analyte, a recovery assay with morphine-D3 was also performed and a similar recovery value was obtained (45% ± 1). Satisfactory results of precision were obtained at the two concentration levels evaluated because the RSD values obtained were lower than 6 and 11% for intra-day and inter-day precision, respectively (Table 2). For this reason, morphine and oripavine also could be quantified in real samples using a correction factor. According to SANTE/11813/2017, the recoveries between 30 and 70% and 120 and 140% can be acceptable if RSD is ≤20% [35].

**Figure 5:**
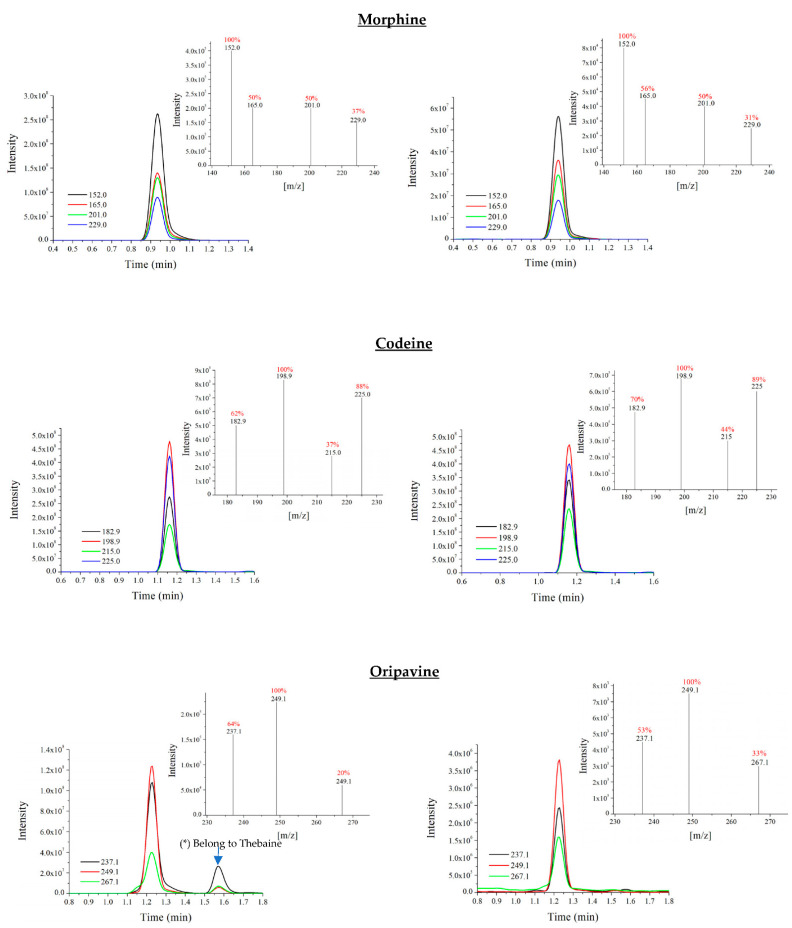

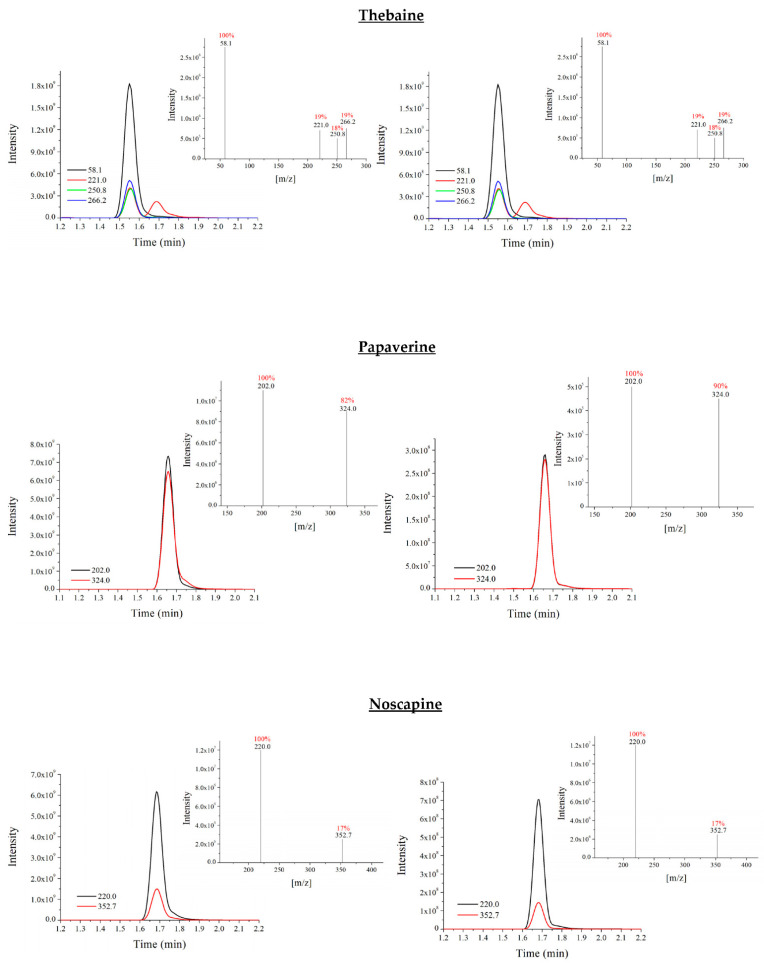
Comparison between the extracted ions chromatograms and the intensity of fragment ions mass spectrum (relative abundance (%) in colour red) obtained for each of the compounds in a standard solution mixture 1 mg/L (**left**) with respect to a poppy seed sample (**right**).

A good selectivity of the method was obtained as shown in Figure 5. When comparing the chromatograms and the spectra of each of the analytes, it was found that the variation of the retention time was in all cases ≤ 0.1 min deviation and the ion ratios of the sample extracts were within ±30% (relative) of the average of the calibration standards for each of the analytes, which is in accordance with established in the reference guide [35]. All samples showed two peaks to codeine. However, the first peak did not coincide with the retention time of the standard and the ratio of the product ions did not accord. As it did not comply with these two criteria, this first peak was discarded and was considered not to be codeine; instead, it was considered to be a matrix product. In addition, for oripavine, a second peak was also observed in some standards or samples, which was related to thebaine since it appeared in its retention time, and since oripavine is its main metabolite and some of its transitions were similar.

Finally, these results indicated that the proposed method showed good analytical performance and could be successfully used for the extraction, purification by MSPE and quantification of poppy seeds samples.

### 3.5. Comparison of the Proposed Methodology with Others Reported Methods

The developed method was compared with other methods published in recent years for the analysis of opium alkaloids in poppy seeds, straw or capsules and hot pot, a traditional Chinese food. Table 3 summarises the extraction, purification and analytical techniques used and the main characteristics of each methodology. As has been observed, the most used separation technique is HPLC, although there are authors who also use UHPLC because it has the advantage of reducing analysis times. Chromatography times for methods developed with HPLC-MS/MS are between 15 and 30 min [6,20], while methods developed with UHPLC-MS/MS take between 7 and 10 min [1,22]. A noteworthy aspect of this proposed method is that only 5 min were sufficient to efficiently quantify the six opium alkaloids, which is considerably less time than the methods already developed to quantify opioids in the literature. This is very important since reducing the analysis time also reduces the cost. In addition, the detector that has been mainly used is the QqQ, which means a much more selective detection with higher sensitivity, although there are some studies with other detectors, such as DAD or UV detector [16,24].

As can be seen in Table 3, the main extraction technique used in the literature is SLE with organic solvents (acetonitrile/water/formic acid, acidified methanol or pure methanol) [1,6,17]. The main disadvantage of these methods is the high consumption of organic solvents, which is about 200 mL. This is a very important aspect to consider as they are damaging to the environment. In addition, although it provides some good recovery results, it can result in incorrect results due to matrix interferences, as seen in this work and the comments of some of the authors in their works [1]. Furthermore, it should be considered that since there are so many impurities that have not been removed, the chromatographic column and the instrument become dirty more easily, which shortens its life of use. Therefore, it is very important to perform a purification step that can remove these matrix interferences. The conventional purification technique and which two authors use with these analytes is SPE [19,20]. However, it is a very complex technique to operate compared to other new purification techniques, such as MSPE, because it avoids the strict control of the loading and elution flow rate and obstruction problems. There are still few authors who have started to use magnetic adsorbent materials to purify samples to quantify opioids and none of them uses it for poppy seeds samples. Three have been found in the literature, although one of them only analyses two of the main opioids. In all of them, minimisation of matrix interferences and good recoveries has been obtained by selectively adsorbing the analytes. However, in the methodology proposed in this work, lower MDL and MQL have been obtained than in other published works (between 0.07–72.01 µg/kg and 0.24–240 µg/kg, respectively). In addition, the advantage of this method over others is that purification is completed in only 4 min (1 min of adsorption and 3 min of desorption), unlike others that take up to 30 min [23]. Moreover, it is the first methodology developed and validated for the quantification of the six main opium alkaloids in poppy seeds, which is very important to take into account since EFSA claimed, in its last opinion in 2018, that there was an absence of studies with all these analytes and, therefore, there is a need to develop methodologies capable of quantifying all of them [15]. The result is that this method is completely able, in a quick, easy and efficient manner, to quantify the six target analytes in poppy seed samples.

### 3.6. Application of the Method SLE-MSPE-UHPLC-QqQ-MS/MS to Real Samples of Poppy Seeds

Finally, the method was applied to analyse 16 seeds samples: 11 commercial poppy seeds, 2 wild poppy seeds and 3 commercial edible seeds mix (Table S1). Once the areas of each of the samples were obtained, the corresponding recovery values (Table 2) were applied and these results were divided by the areas obtained from the internal standard (morphine-D3). Finally, they were interpolated into matrix-matched calibration curves with morphine-D3 to minimise the possible signal errors and to produce more accurate results.

All samples were found to contain the six opium alkaloids (Table 4) and so they were contaminated by latex from the *Papaver somniferum* L. plant. This result was very surprising because three of them were labelled as being from *Papaver rhoeas* L. species, which does not contain opium alkaloids (samples PS03, PS05 and PS06). Moreover, physically, they look similar to the blue poppy seeds of the *Papaver somniferum* L. species. Therefore, these two aspects provided enough evidence to assert that there was incorrect labelling of these products. For comparison, wild corn poppy seeds (PS10), which were black and small, were analysed and none of the analytes was detected, whereas in wild opium poppy seeds (PS9), the six target analytes were found. This confirms the theory that the common poppy (*Papaver rhoeas* L.) does not contain opium alkaloids and the labels of the commercial products analysed were incorrect.

In addition, the concentrations of each type of poppy seed were calculated. Due to the surface contamination and the large number of other factors influencing the concentration of these alkaloids in poppy seeds, variable amounts have been found in the same batch of seeds. For these reasons, the range of concentration is provided to show the extent of the variation and is in accordance with other researchers who identified a variation in opium alkaloids within a batch and between batches of poppy seeds analysed [1,2,44]. Concentrations of morphine ranged from 1.5 to 249.0 mg/kg, codeine from <0.2 µg/kg to 45.8 mg/kg, thebaine from <2.4 µg/kg to 136.2 mg/kg, papaverine from <0.2 µg/kg to 27.1 mg/kg, noscapine from <0.2 µg/kg to 108.7 mg/kg and oripavine from <240 µg/kg to 33.4 mg/kg. The opium alkaloid concentrations obtained in poppy seeds agreed with concentrations determined by other researchers. In the case of morphine, wide ranges of concentrations were obtained by López et al.; 0.2–241 mg/kg, Sproll et al.; between <1 and 270 mg/kg, Bjerver et al. who found 2.6–106.7 mg/kg; and Hayes et al. between 17 and 294 mg/kg [1,6,45,46]. Lower results were found by Carlin et al. between 3 and 64 mg/kg [44] and higher levels were found by Powers et al. from <1 to 2788 mg/kg [3]. Regarding codeine, the results obtained were very similar to the results obtained by Sproll et al., who found <0.3–56 mg/kg [6] but were lower than the results found by López et al. who found from <0.1 to 348 mg/kg and Powers et al. from <1 to 247.6 mg/kg [1,3]. On the other hand, the results obtained for thebaine were similar to those of López et al., as they found <0.1–106 mg/kg and Powers et al. from <1 to 124 mg/kg [1,3]. Regarding papaverine and noscapine, the contents found in this study were higher than others, as Sproll et al. did not detect their presence in any of their samples [6] and López et al. only determined <0.1–3.8 mg/kg of papaverine and <0.1–5 mg/kg of noscapine [1]. Notably, oripavine had not been previously analysed in poppy seeds by any author.

In addition, many of the samples showed values below the MQL but in all of them considerably high values of morphine were quantified, which meant that all samples except two (87%) exceeded the maximum limit established by Germany (4 mg/kg) and the 73% exceeded the reference level established in the EU (10 mg/kg morphine in poppy seeds for direct human consumption). It therefore implies that this agreement is not being fulfilled. In addition, 73% exceeded the Hungarian limit for morphine (30 mg/kg). For the other analytes, the maximum limit established by Hungary was 20 mg/kg, so two samples exceeded the maximum limit for codeine (PS02 and PS03), two samples for thebaine (PS03 and PS11), three for papaverine (PS01, PS02 and PS09) and seven for noscapine (PS01, PS02, PS03, PS07, PS08, PS09 and PS11). If oripavine with the same concentration was considered, two samples would exceed this limit (PS02 and PS03). As it can be seen in Table 4, three seed mixes consisting of sunflower, sesame, brown flax, pumpkin and poppy seeds were also analysed. In one of them (MIX2), a considerably high amount of morphine was found (54 mg/kg), taking into account that only 5–10% of the seeds in the mix were poppy seeds. Therefore, poppy seeds used in MIX2 also exceeded the limit maximum established in the EU. All these results confirm the current problems with the marketing of such products in the EU because, in the absence of common legislation, a high number of RASFF health alerts are occurring, some 30 notifications since 2005 [4].

**Figure 6:**
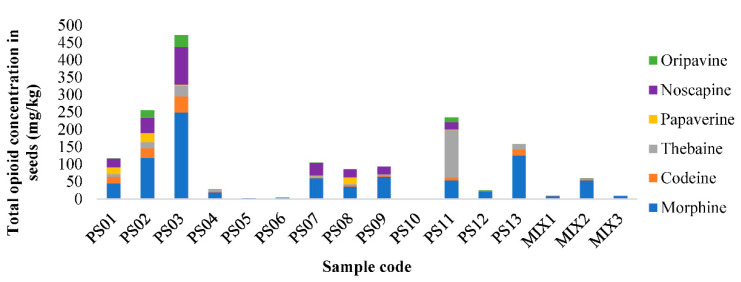
Total content of opium alkaloids found in poppy seeds and seed mixes analysed by the SLE-MSPE-UHPLC-QqQ-MS/MS method proposed.

Considering the highest concentration obtained in each of the batches as shown in Figure 6, the samples with the highest number of total opiate alkaloids are PS03, with approximately 475 mg/kg (supposedly of the *Papaver rhoeas* L. species) and PS02 with approximately 250 mg/kg (physically blue poppy seed from Turkey). Especially high was the amount of thebaine found in PS11 (physically blue poppy seed from Czech Republic). Oripavine was found in all the commercial samples.

## 4. Conclusions

A rapid, simple and efficient method was developed for the prior determination of morphine, codeine, thebaine, papaverine, noscapine and oripavine in poppy seeds by ultra-high-performance liquid chromatography-tandem mass spectrometry. Mesostructured silica-coated magnetic nanoparticles were used as adsorbent for MSPE and the sample extracts were purified in just 4 min. Furthermore, the chromatographic method was only 5 min long, which is very fast and considerably reduces the time and therefore the cost of the analysis. The method was successfully applied to the analysis of commercial poppy seed samples and seed mixes purchased in Spanish supermarkets at the end of 2020. The most surprising result was that incorrect labelling is occurring and so the correct naming of the seeds used is necessary. The six opium alkaloids were found in all the commercial samples analysed and 73% exceeded the reference level for morphine in poppy seeds for direct human consumption established in the EU (<10 mg/kg). All these results confirmed the need to further study the actual exposure to opioid alkaloids of the population by examining not only the consumption of poppy seeds but also other foods containing poppy seeds in order to establish harmonised legislation in all EU members and thus facilitate the common market by decreasing the number of RASFF alerts.

## Figures and Tables

**Table 1:** Textural properties of the six magnetic materials synthesised.

Material	S_BET_ (m^2^/g) ^a^	Pore Volume (cm^3^/g) ^b^	Pore Diameter (Å) ^c^	Elemental Analysis (%)			
				C	N	H	(mmol ligand/g) ^d^
Fe_3_O_4_	-	-	-	-	-	-	-
Fe_3_O_4_@SiO_2_	147	0.18	125.8	-	-	-	-
Fe_3_O_4_@SiO_2_@C_8_	26	0.09	124.4	3.432	0.000	1.070	0.286
Fe_3_O_4_@SiO_2_@C_18_	24	0.10	124.9	3.227	0.000	0.928	0.134
Fe_3_O_4_@SiO_2_@mSiO_2_	355	0.23	38.9	-	-	-	-
Fe_3_O_4_@SiO_2_@mSiO_2_@C_8_	191	0.14	39.0	10.426	0.000	2.174	0.869
Fe_3_O_4_@SiO_2_@mSiO_2_@C_18_	14	0.04	36.4	11.310	0.000	2.178	0.471

^a^ S_BET_: Specific surface area calculated by Brunauer–Emmett–Teller (BET) method. ^b^ Total pore volume was measured at relative pressure (P/P_0_) = 0.97. ^c^ Pore diameter estimated by using the BJH (Barrett, Joyner and Halenda) model applied on the desorption Branch. ^d^ mmol of ligand/g of material calculated with the % obtained by elemental analysis.

**Table 2:** Validation parameters of the SLE-MSPE-UHPLC-QqQ-MS/MS method for the determination of six opium alkaloids in poppy seeds samples.

Analytes	Linear Range (µg/L)	Matrix-Matched Calibration (R^2^)	Cm	ME	MDLn (µg/kg)	MQL (µg/kg)	Accuracy		Precision	
							Recovery (% ± SD)	Mean Recovery (% ± SD)	Intra-Day Precision (RSD %)	Inter-Day Precision (RSD %)
Morphine	0.01–5000	y = 3269x + 32,452 (0.999)	92	64	0.07	0.24	50 ± 1 ^a^	46 ± 2	4 ^a^	11 ^a^
							42 ± 2 ^b^		2 ^b^	5 ^b^
Codeine	0.01–5000	y = 2554x + 29,642 (0.999)	96	86	0.07	0.24	64 ± 2 ^a^	68 ± 4	3 ^a^	8 ^a^
							71 ± 6 ^b^		2 ^b^	4 ^b^
Thebaine	0.1–5000	y = 10,093x + 485,953 (0.999)	96	109	0.72	2.40	72 ± 3 ^a^	74 ± 4	5 ^a^	7 ^a^
							76 ± 4 ^b^		0 ^b^	4 ^b^
Papaverine	0.01–5000	y = 33,441x + 2,814,320 (0.999)	92	80	0.07	0.24	116 ± 5 ^a^	109 ± 3	5 ^a^	9 ^a^
							101 ± 1 ^b^		2 ^b^	7 ^b^
Noscapine	0.01–5000	y = 42,914x + 1,631,091 (0.999)	94	65	0.07	0.24	109 ± 1 ^a^	103 ± 1	4 ^a^	10 ^a^
							97 ± 1 ^b^		3 ^b^	8 ^b^
Oripavine	10–5000	y = 1080x − 31,137 (0.999)	94	31	72.07	240	51 ± 3 ^a^	52 ± 3	3 ^a^	5 ^a^
							53 ± 3 ^b^		3 ^b^	5 ^b^

Cm: linearity coefficient calculated by (1-(SD/average slope)) × 100, where SD is the standard deviation of the calibration slopes obtained on different days; ME: matrix effect calculated by dividing the purified matrix slope by the solvent slope. MDL: method detection limit. MQL: method quantification limit estimated as 3 and 10 times the signal/noise ratio, respectively. Accuracy (mean recovery obtained from six samples, *n* = 6) and precision were obtained by spiking samples at two known concentration levels: ^a^ low spiked level (0.1 mg/kg) and ^b^ high spiked level (1 mg/kg). Intra-day precision: six consecutive injections (*n* = 6) on the same day; Inter-day precision: three replicate samples injected in triplicate throughout three different days (*n* = 9).

**Table 3:** Comparison of the developed SLE-MSPE-UHPLC-QqQ-MS/MS method with other reported methods for the detection of opium alkaloids in poppy (seeds, straw or capsules) and hot pot.

Sample	Analytes	Sample Treatment		Analysis Technique	MDL (µg/kg)	MQL (µg/kg)	Recovery (%)	RSD (%)	Ref.
		Extraction	Purification						
Poppy seeds	MOR, COD, THEB, NOS, PAP	AcN/water/formic acid, 80/19/1, *v/v/v* (100 mL, 30 min, ×2)	−	UHPLC-QqQ-MS/MS	−	100	77–172	<20.0	[1]
Poppy seeds, cake, buns	MOR, COD, PAP, NOS	MeOH 0.1% acetic acid (30 mL, 60 min)	−	HPLC-QqQ-MS/MS	70–300	200–1000	−	7.4–9.0	[6]
Poppy straw	MOR, COD, THEB, PAP	MeOH (5 mL, 20 min, ×2)	−	HPLC-DAD	200–1800	600–5400	97–99	0.2–0.4	[17]
Hot pot	MOR, COD, THEB, PAP, NOS	HCl 0.1 M (20 mL, 10 min) and PE (10 mL)	SPE (Oasis MCX 60 mg)	UHPLC-QqQ-MS/MS	0.003–0.04	0.01–0.1	72–124	7.9–23.7	[19]
Poppy straw	MOR, COD, THEB, PAP	Water 5% acetic acid	SPE (Oasis MCX)	HPLC- Ion trap-MS/MS	400–17,500	1100–52,200	−	−	[20]
Hot pot	MOR, COD, THEB, PAP, NOS	Water/AcN 50% (20 mg, 5 min)	MSPE (Fe_3_O_4_@SiO_2_@ADME 50 mg)	HPLC-QqQLIT-MS/MS	0.05–0.8	0.25–2.5	80–115	4.3–10.7	[22]
Hot pot	MOR, COD, THEB, PAP, NAR	AcN 0.1% formic acid and n-hexane	MSPE (Fe_3_O_4_@SiO_2_@CS/GO 15 mg)	UHPLC- QqQLIT-MS/MS	0.016–0.092	0.036–0.31	75–104	0.7–9.5	[23]
Poppy capsules	NAR, PAP	SFE	MSPE (Fe_3_O_4_@Cu@DPTC 50 mg)	HPLC-UV	1–100	−	88–99	5.8–7.7	[25]
Poppy seeds	MOR, COD, THEB, PAP, NOS, ORIP	MeOH/water, 50/50 (*v/v*)	MSPE (Fe_3_O_4_@SiO_2_@mSiO_2_ 50 mg)	UHPLC-QqQ-MS/MS	0.07–72.01	0.24–240	46–109	0.4–11	This work

MOR: morphine, COD: codeine, THEB: thebaine, PAP: papaverine, NOS: noscapine, NAR: narceine, ORIP: oripavine, AcN: acetonitrile, MeOH: methanol, HCl: hydrochloride acid, PE: petroleum ether, SFE: supercritical fluid extraction, SPE: solid phase extraction, MCX: mixed-mode, strong cation-exchange, MSPE: magnetic solid phase extraction, DPTC: diphenylthiocarbazone, (U)HPLC: (ultra)-high performance liquid chromatography, QqQ: triple quadrupole, MS/MS: tandem mass spectrometry, DAD: diode-array detector, QqQLIT: quadrupole linear ion trap, UV: ultraviolet detector, MDL: method detection limit, MQL: method quantification limit, RSD: relative standard deviation.

**Table 4:** Range of occurrence (mg/kg) of each of the six opium alkaloids analysed in three samples (*n* = 3).

Code	Morphine	Codeine	Thebaine	Papaverine	Noscapine	Oripavine
PS01	20.6–45.4	5.7–19.2	<MQL–7.3	7.3–19.5	9.4–24.8	<MQL–0.6
PS02	23.2–118.7	3.2–26.7	0.9–17.7	2.1–27.1	6.5–42.6	3.8–22.9
PS03	154.9–249.0	21.3–45.8	12.4–31.5	0.8–2.9	0.3–108.7	9.8–33.4
PS04	17.0–19.5	1.0–1.9	3.4–6.5	<MQL	<MQL	<MQL
PS05	1.9–2.2	<MQL	<MQL	<MQL	<MQL	<MQL
PS06	1.5–3.7	<MQL	<MQL	<MQL	<MQL	<MQL
PS07	6.9–59.3	0.4–0.9	<MQL–5.8	<MQL–1.1	0.7–36.3	<MQL–0.8
PS08	28.8–35.1	3.4–4.0	0.8–3.4	10.5–19.9	18.1–22.7	<MQL
PS09	58.6–64.3	2.9–3.6	1.3–3.2	<MQL	19.0–21.7	<MQL
PS10	ND	ND	ND	ND	ND	ND
PS11	33.7–53.0	3.7–8.7	37.4–136.2	<MQL–1.9	2.0–21.1	2.3–13.8
PS12	4.1–22.3	<MQL-0.3	<MQL-1.5	<MQL	<MQL	<MQL-0.5
PS13	49.6–125.4	2.3–16.5	3.9–15.6	<MQL	<MQL	<MQL-0.5
MIX1	6.5–8.7	<MQL-0.04	<MQL	<MQL	<MQL	<MQL
MIX2	44.0–54.0	0.7–3.0	0.68–2.6	<MQL	<MQL	<MQL-0.3
MIX3	7.0–8.6	<MQL	<MQL	<MQL	<MQL	<MQL

MQL: method quantification limit (for codeine, papaverine and noscapine: 0.24 µg/kg; for thebaine: 2.40 µg/kg; and for oripavine: 240 µg/kg). ND: not detected. To quantify seed samples, matrix-matched calibration was used with internal standard (morphine: y = 0.006x − 0.182; codeine: y = 0.005x + 0.117; thebaine: y = 0.020x + 1.853; papaverine: y = 0.069x + 6.739; noscapine: y = 0.080x + 6.049; oripavine: y = 0.002x + 0.009).

## Supplementary Materials

The following are available online at https://www.mdpi.com/article/10.3390/foods10071587/s1, Section S1.1: Instrumental and Method Validation, Figure S1: SEM images of Fe_3_O_4_ (**a**), Fe_3_O_4_@SiO_2_ (**b**) and Fe_3_O_4_@SiO_2_@mSiO_2_ (**c**) magnetic particles, Figure S2: XRD patterns of at low angles (**a**) and wide angles (**b**): Fe_3_O_4_ (1), Fe_3_O_4_@SiO_2_ (2), Fe_3_O_4_@SiO_2_@C_8_ (3), Fe_3_O_4_@SiO_2_@C_18_ (4), Fe_3_O_4_@SiO_2_@mSiO_2_ (5), Fe_3_O_4_@SiO_2_@mSiO_2_@C_8_ (6) and Fe_3_O_4_@SiO_2_@mSiO_2_@C_18_ (7), Figure S3: FT-IR spectra of particles with different treatment to remove the surfactant (**a**): calcination treatment Fe3O4@SiO_2_@mSiO_2_ (1) and particles after treatment with acetone extraction Fe_3_O_4_@SiO_2_@CTAB/mSiO_2_ (2). FT-IR spectra of 7 synthesised materials (**b**): Fe_3_O_4_ (1), Fe_3_O_4_@SiO_2_ (2), Fe_3_O_4_@SiO_2_@mSiO_2_ (3), Fe_3_O_4_@SiO_2_@C_8_ (4), Fe_3_O_4_@SiO_2_@C_18_ (5), Fe_3_O_4_@SiO_2_@mSiO_2_@C_8_ (6) and Fe_3_O_4_@SiO_2_@mSiO_2_@C_18_ (7), Figure S4: Image of the first three washes of the Fe_3_O_4_@SiO_2_@mSiO_2_ material by magnetic decantation, Figure S5: N_2_ adsorption–desorption isotherms (**a**,**c**) and pore-size distribution (**b**,**d**) of Fe_3_O_4_@SiO_2_ and Fe_3_O_4_@SiO_2_@mSiO_2_, respectively, before and after the modification with C_8_ and C_18_ ligands, Figure S6: Extracted areas of target analytes in 2.5 g poppy seeds and 10 mL of solvent during 10 min under each of the extraction conditions to be optimised (extraction solvent and agitation type), Figure S7: Extracted areas of the six target analytes in 2.5 g of poppy seeds with methanol/water 50/50 (*v/v*) and magnetic stirring under each of the double extraction conditions (volume and time), Figure S8: Extracted areas of the six target analytes in 2.5 g of poppy seeds with methanol/water 50/50 (*v/v*), magnetic stirring and double extraction of 30 mL for 30 min under each of the solvent pH values, Figure S9: Adsorption kinetic (**a**) and isotherm (**b**) experiments of six opium alkaloids with 100 mg Fe_3_O_4_@SiO_2_@mSiO_2_ material, Figure S10: Three kinetics models (**a**) and two isotherm models (**b**) for the adsorption of six opium alkaloids on the Fe_3_O_4_@SiO_2_@mSiO_2,_ Figure S11: Effect of the solvent pH value in the adsorption step in methanol/water 50/50 (*v/v*) with 100 mg of Fe_3_O_4_@SiO_2_@mSiO_2_ material for 1 min, Table S1: Registration of the different poppy seed samples used with their description, species and origin, Table S2: Optimal parameters of MRM for the analysis of each opium alkaloids by UHPLC-QqQ-MS/MS: precursor and fragment ions, cone voltage (CV), collision energy (CE) and retention time (Rt) of each opium alkaloid, Table S3: Equations of adsorption kinetic and isotherm, Table S4: Kinetic parameters of the adsorption of six opioid alkaloids with 100 mg Fe_3_O_4_@SiO_2_@mSiO_2_ material for different times (1–20 min) based on different kinetic models, Table S5: Recovery values (%) ± standard deviation (SD) obtained in the MSPE procedure with the solvents used in the SLE of poppy seeds at different desorption times (1, 5, 10 and 20 min), Table S6: Recovery values (%) ± standard deviation (SD) obtained in MSPE procedure with eight desorption solvents at different pH values (non-modified, acid with 1% formic acid, basic with 1% ammonia and basic with 10% ammonia), Table S7: Recovery values (%) ± standard deviation (SD) obtained in the MSPE procedure with the mixture of the solvents that showed the best results in desorption step with different proportions (50/50, 80/20 and 20/80, *v/v*), all of them at basic pH with solvent/ammonia, 90/10 *v/v*, Table S8: Recovery values (%) ± standard deviation (SD) obtained in MSPE procedure after a different number of consecutive desorptions with diethyl ether/methanol, 80/20, *v/v*, with 10% ammonia at 1 min, Table S9: Instrumental validation parameters (solvent linearity, solvent calibration, LOQ and LOD).
