# Supplementary material for: Mesostructured Silica-Coated Magnetic Nanoparticles to Extract Six Opium Alkaloids in Poppy Seeds Prior to Ultra-High-Performance Liquid Chromatography-Tandem Mass Spectrometry Analysis
Source: Foods. 2021 Jul 8;10(7):1587. doi: 10.3390/foods10071587 (PMC8307897; doi:10.3390/foods10071587)
Supplement: Supplementary file 1 [file foods-10-01587-s001.zip › foods-1264633-supplementary.pdf]

Supplementary Information

S1.1 Instrumental and Method Validation

Firstly, the linearity was determined as the six analytes dissolved in the pure solvent, as in the blank extract after extraction and purification by MSPE. Linear regression analysis was applied, and linearity was expressed as the coefficient of determination (R^2) and the linearity coefficient (Cm). Cm was calculated as (1 – (SD/average slope)) x 100, where SD is the standard deviation of the calibration slopes obtained on different days ($n = 3$). Then, the matrix effect (ME, %) was calculated with the comparison of slope purified matrix versus slope solvent. The ME was considered tolerable from 80 to 120%, less than 80% was considered a signal suppression effect and more than 120% signal enhancement effect. In addition, detection and quantification instrumental and method limits were determined from the analysis of the lowest concentration analysed (0.01 μg/L) and they were estimated as three or ten times the signal/noise ratio (S/N), respectively. Accuracy and precision were evaluated at two different levels of concentration, low (0.1 mg/kg) and high (1 mg/kg). Intra-day precision (repeatability) was evaluated by analysing six times on the same day ($n = 6$) and inter-day precision (reproducibility) was evaluated on three different days by analysing each day three times ($n = 9$). In this case, morphine-D3 was also added as an internal standard to compare its results with those of the other analytes. Finally, selectivity was evaluated through the spectra of the different analytes obtained from standards compared with the spectra obtained from samples. Selectivity was considered satisfactory when the variation between spectra was less than 30% and the retention time interval for each analyte was ± 2.5 % [34].

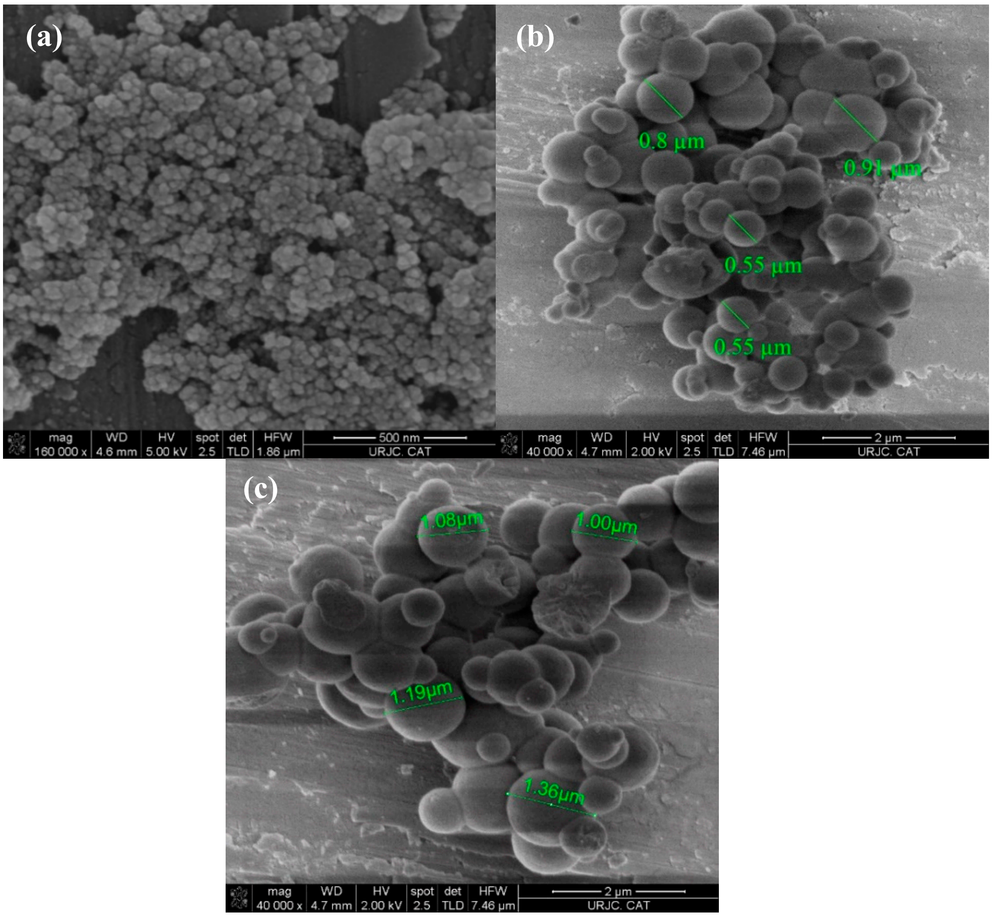

Figure S1. SEM images of Fe_3O_4 (a), $Fe_3O_4@SiO_2$ (b) and $Fe_3O_4@SiO_2@mSiO_2$ (c) magnetic particles.

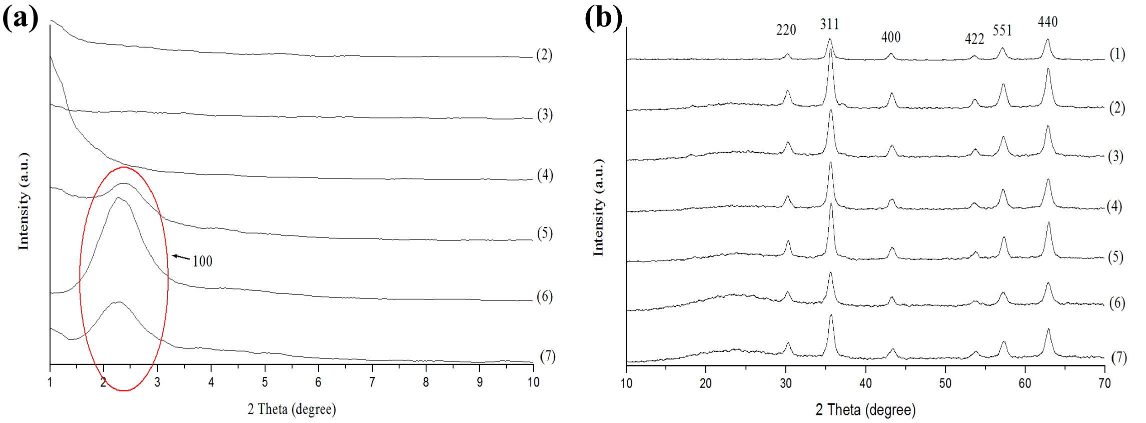

Figure S2. XRD patterns of at low angles (a) and wide angles (b): Fe_3O_4 (1), $Fe_3O_4@SiO_2$ (2), $Fe_3O_4@SiO_2@C_8$ (3), $Fe_3O_4@SiO_2@C_{18}$ (4), $Fe_3O_4@SiO_2@mSiO_2$ (5), $Fe_3O_4@SiO_2@mSiO_2@C_8$ (6) and $Fe_3O_4@SiO_2@mSiO_2@C_{18}$ (7).

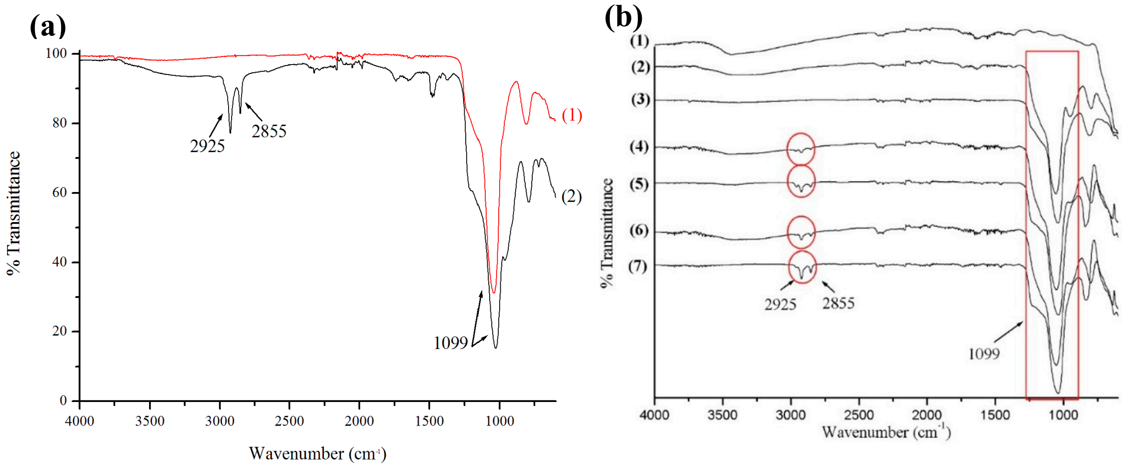

Figure S3. FT-IR spectra of particles with different treatment to remove the surfactant (a): calcination treatment $Fe_3O_4@SiO_2@mSiO_2$ (1) and particles after treatment with acetone extraction $Fe_3O_4@SiO_2@CTAB/mSiO_2$ (2). FT-IR spectra of 7 synthetized materials (b): Fe_3O_4 (1), $Fe_3O_4@SiO_2$ (2), $Fe_3O_4@SiO_2@mSiO_2$ (3), $Fe_3O_4@SiO_2@C_8$ (4), $Fe_3O_4@SiO_2@C_{18}$ (5), $Fe_3O_4@SiO_2@mSiO_2@C_8$ (6) and $Fe_3O_4@SiO_2@mSiO_2@C_{18}$ (7).

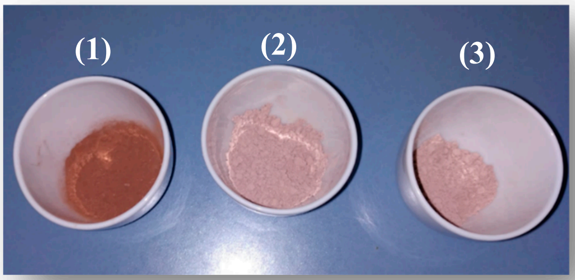

Figure S4. Image of the first three washes of the $Fe_3O_4@SiO_2@mSiO_2$ material by magnetic decantation: first wash (1), second wash (2) and third wash (3).

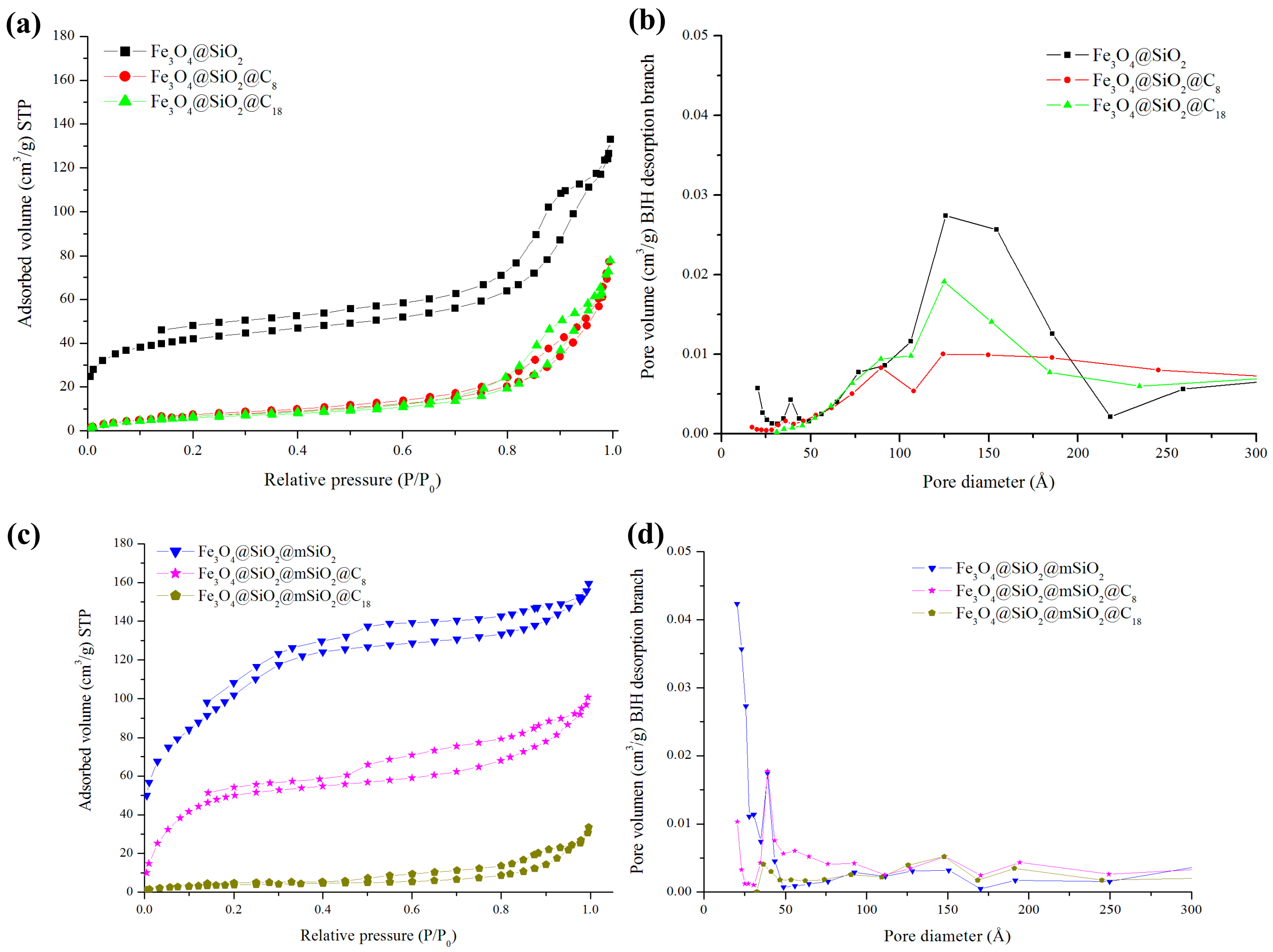

Figure S5. N_2 adsorption-desorption isotherms (a, c) and pore-size distribution (b, d) of $Fe_3O_4@SiO_2$ and $Fe_3O_4@SiO_2@mSiO_2$, respectively, before and after the modification with C_8 and C_{18} ligands.

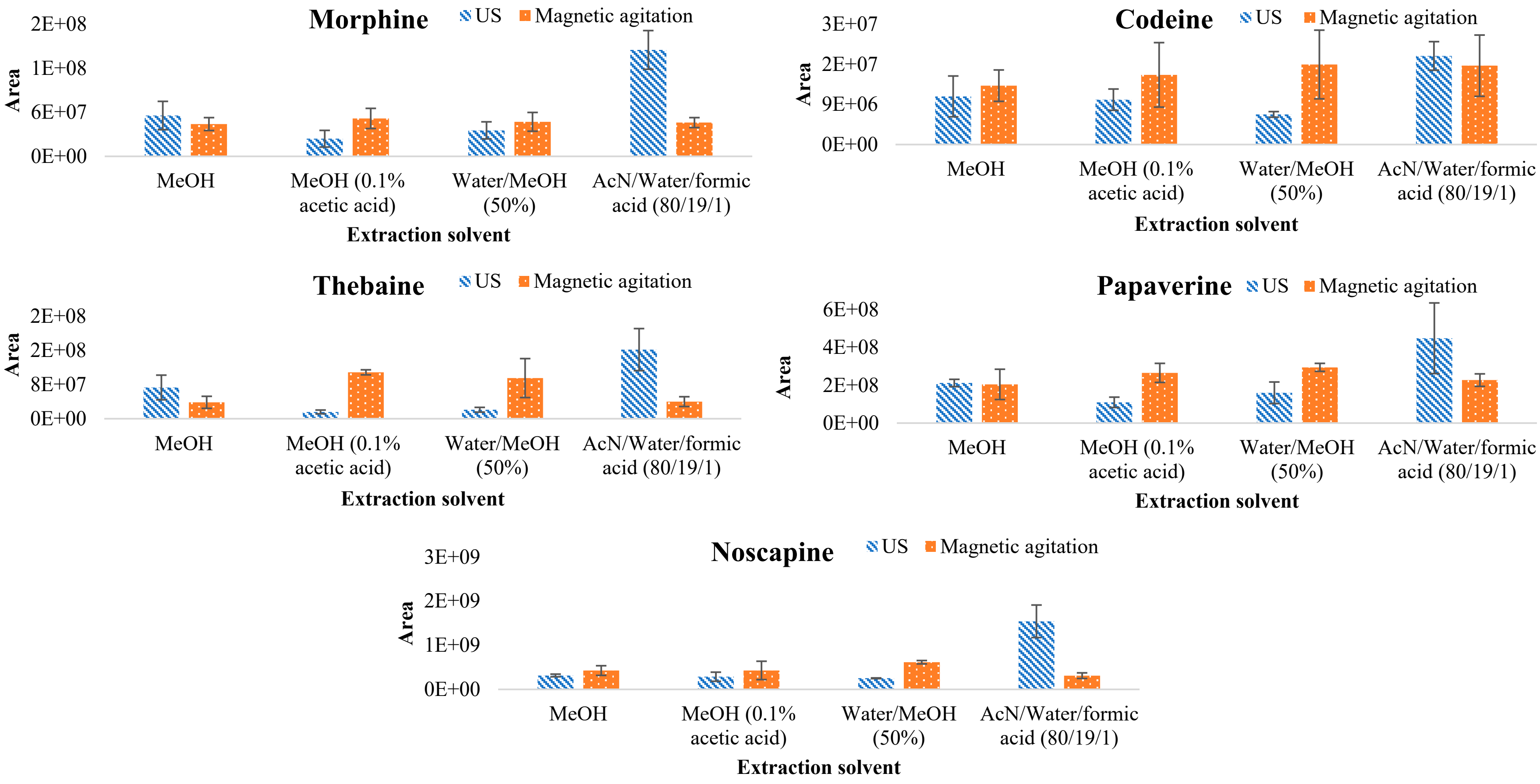

Figure S6. Extracted areas of target analytes in 2.5 g poppy seeds and 10 mL solvent during 10 min under each of the extraction conditions to be optimised (extraction solvent and agitation type).

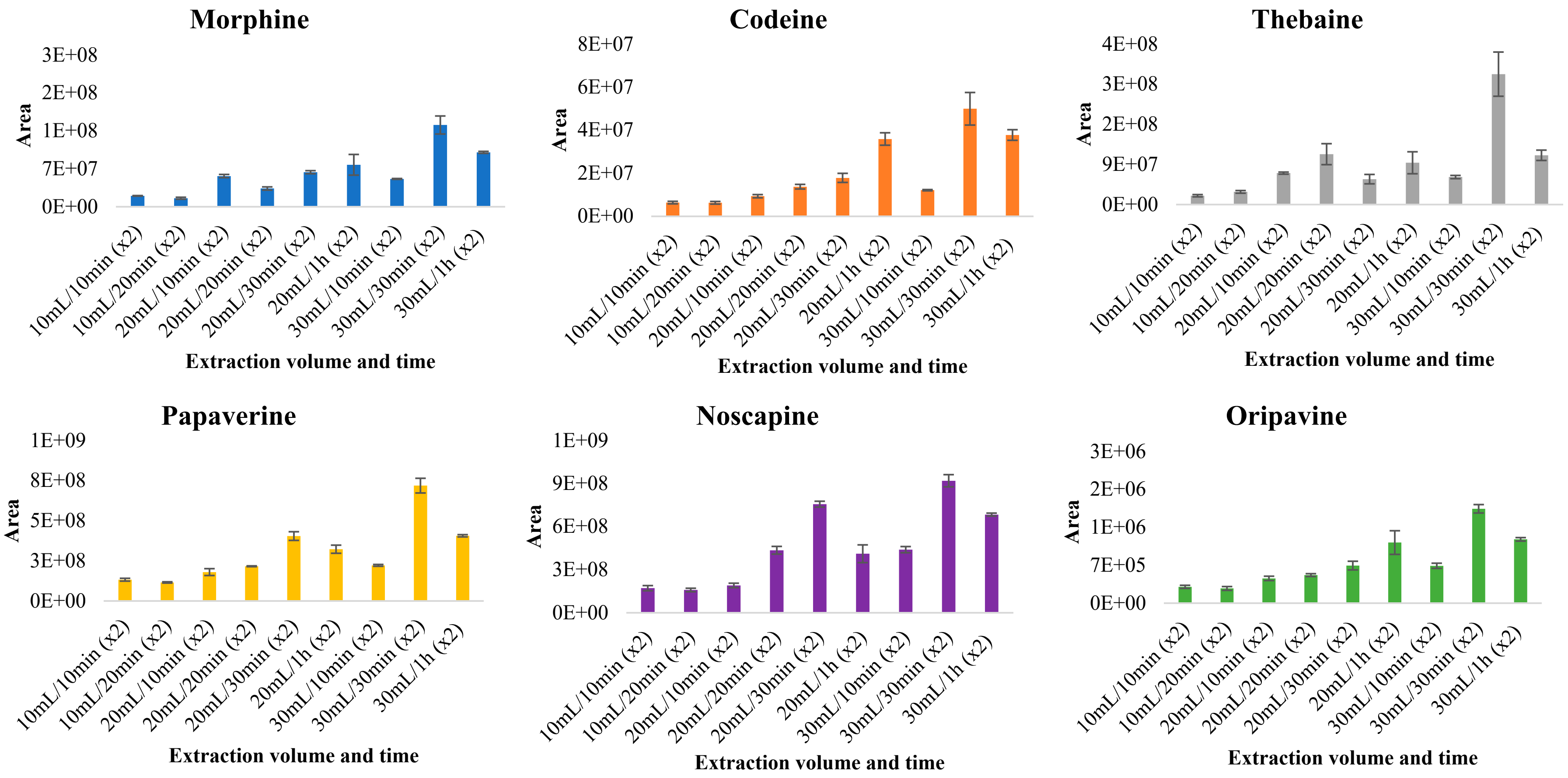

Figure S7. Extracted areas of the 6 target analytes in 2.5 g of poppy seeds with methanol/water 50/50 (v/v) and magnetic stirring under each of the double extraction conditions (volume and time).

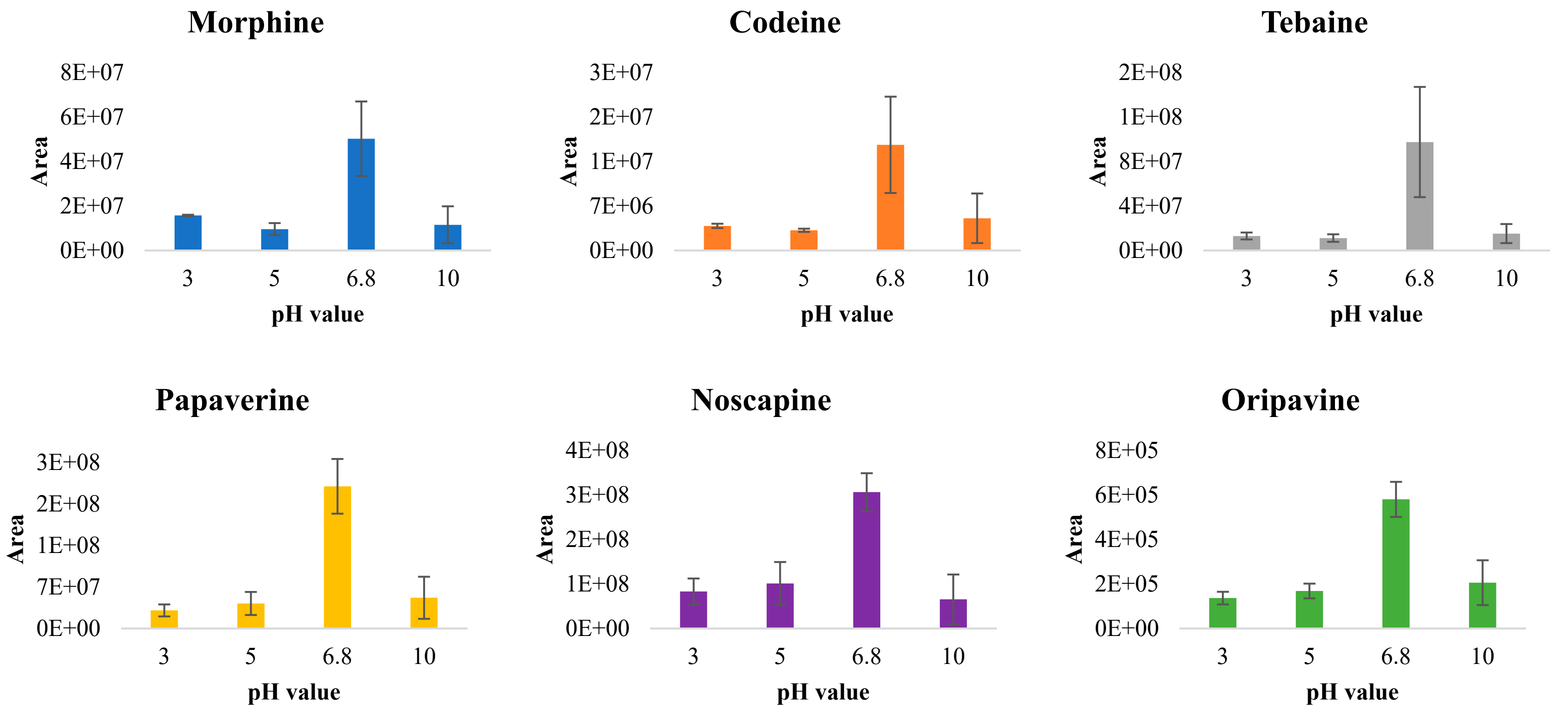

Figure S8. Extracted areas of the 6 target analytes in 2.5 g of poppy seeds with methanol/water 50/50 (v/v), magnetic stirring and double extraction of 30 mL for 30 min under each of the solvent pH values.

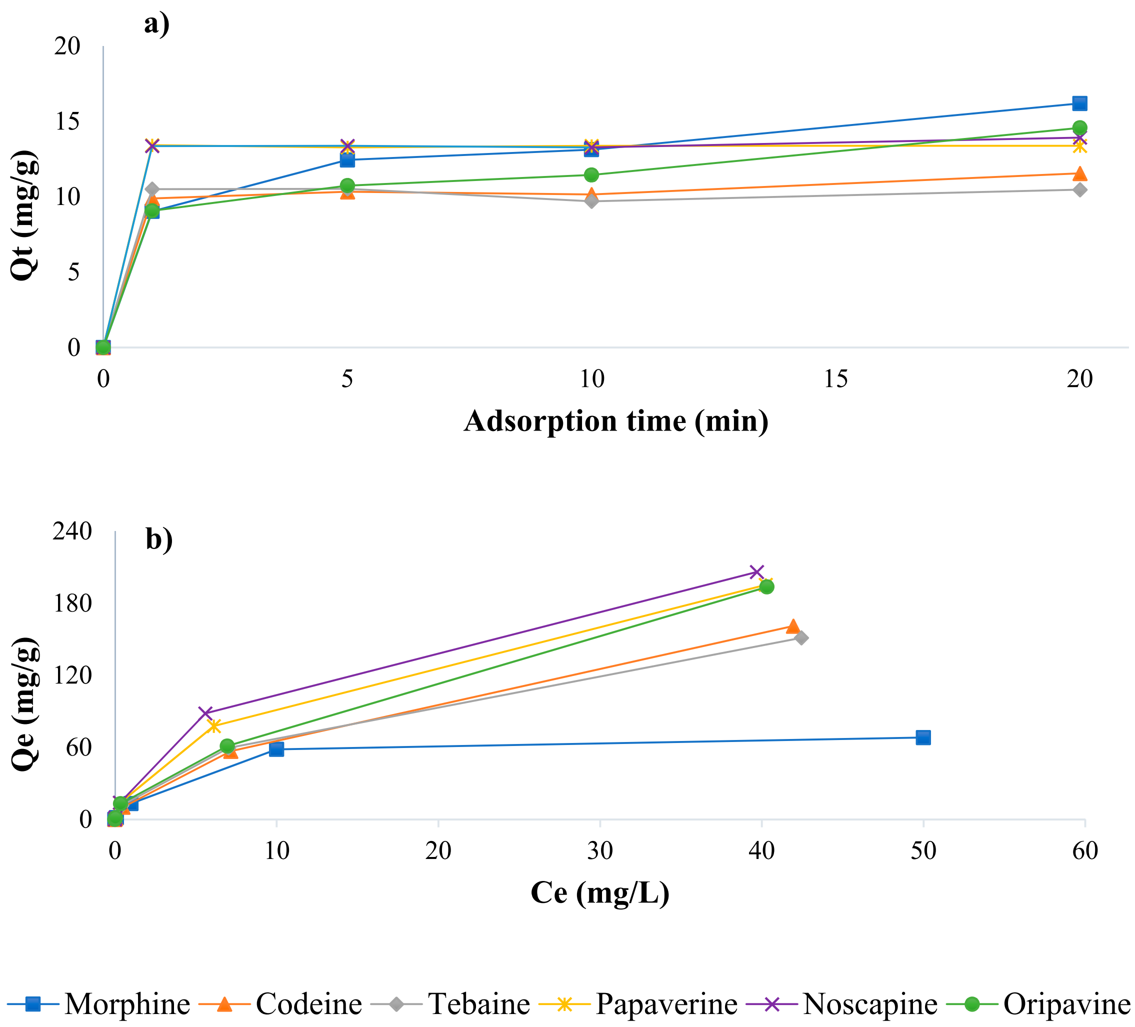

Figure S9. Adsorption kinetic (a) and isotherm (b) experiments of six opium alkaloids with 100 mg Fe_3O_4@SiO_2@mSiO_2 material.

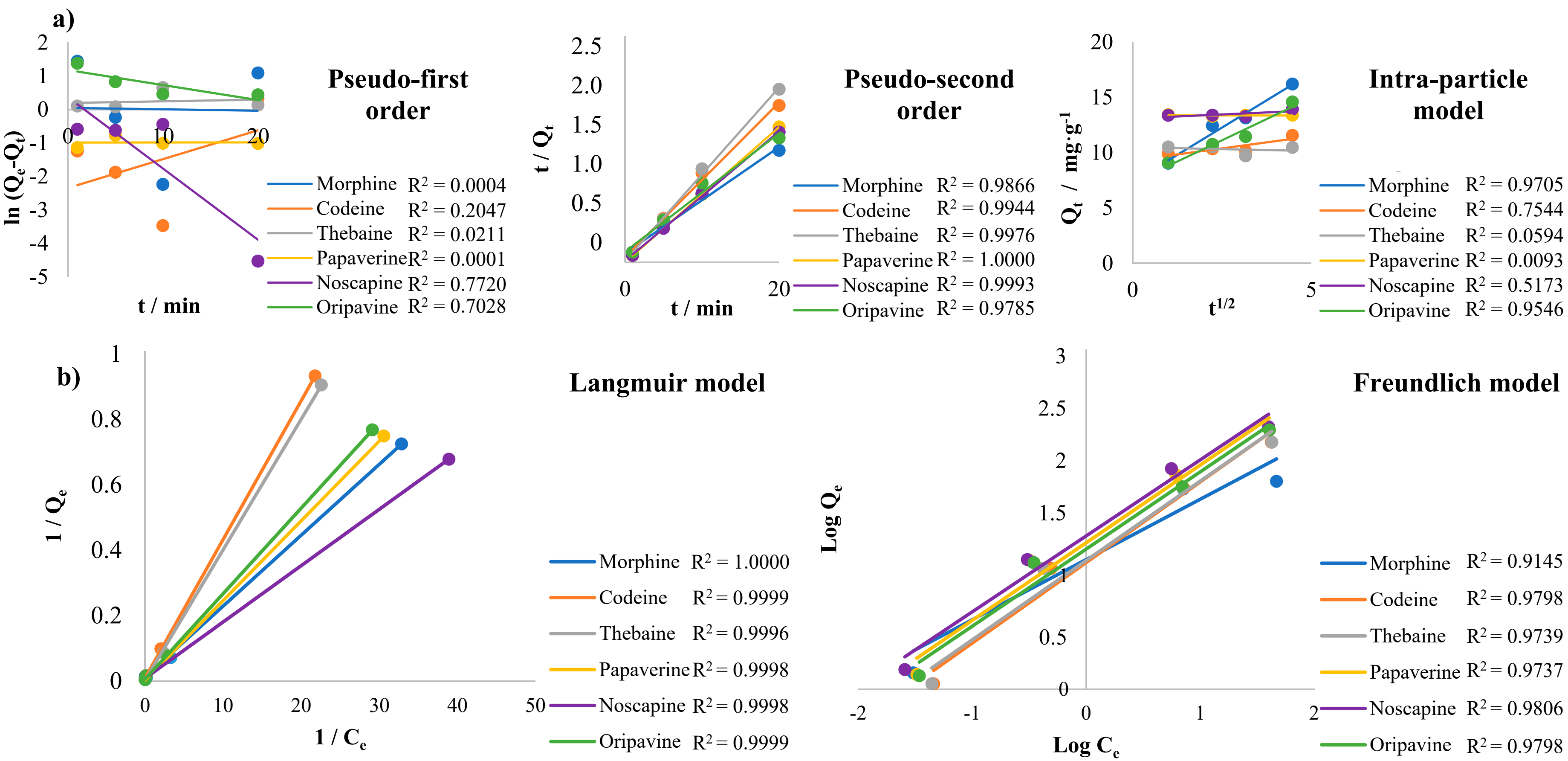

Figure S10. Three kinetics models (a) and two isotherm models (b) for the adsorption of six opium alkaloids on the Fe_3O_4@SiO_2@mSiO_2.

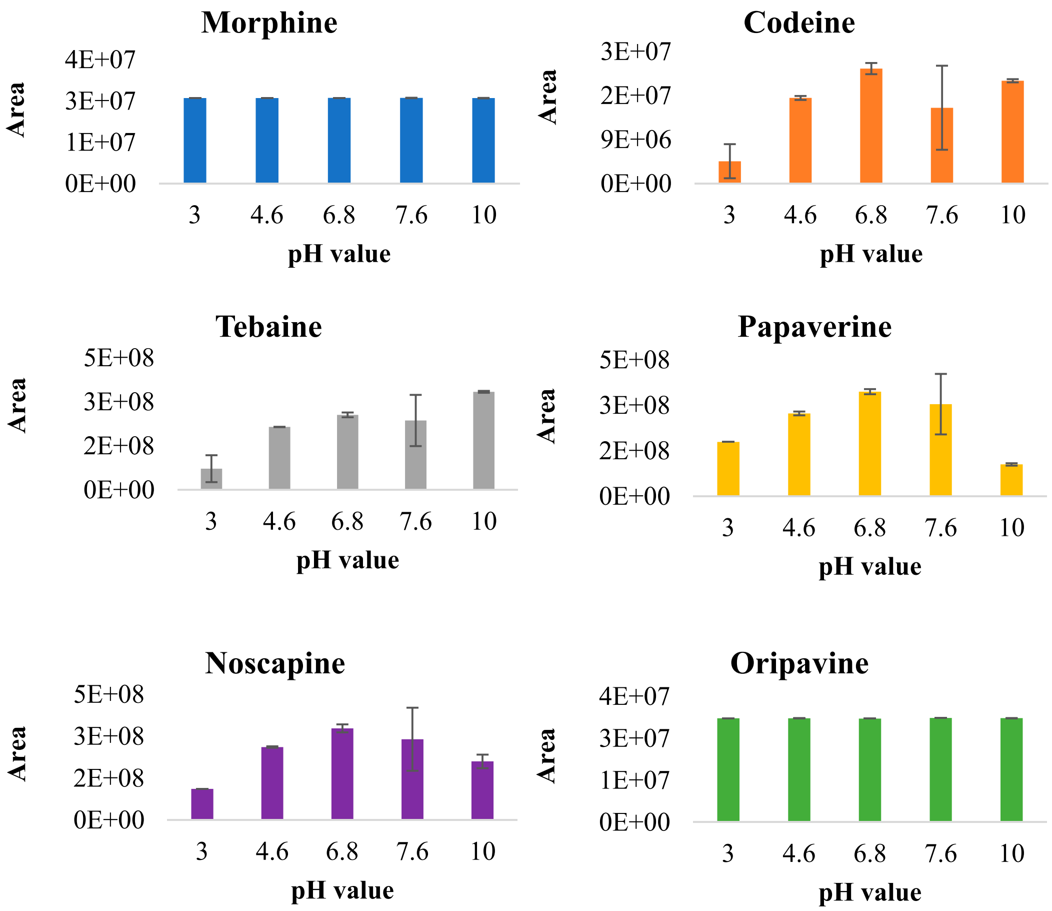

Figure S11. Effect of the solvent pH value in the adsorption step in methanol/water 50/50 (v/v) with 100 mg of $Fe_3O_4@SiO_2@mSiO_2$ material for 1 min.

Table S1. Registration of the different poppy seed samples used with their description, species and origin.

Code	Description	Species specified by labelling	E/No-E	Origin
PS01	Blue poppy seeds	*P. somniferum*	E	Turkey
PS02	Not specific on the label (physically: blue poppy seeds)	Unknown	E	Turkey
PS03	Physically: blue poppy seeds	*P. rhoeas*	No-E	Unknown
PS04	Not specific on the label (physically: blue poppy seeds)	Unknown	No-E	Spain
PS05	Physically: blue poppy seeds	*P. rhoeas*	No-E	Unknown
PS06	Physically: blue poppy seeds	*P. rhoeas*	No-E	Unknown
PS07	Blue poppy seeds	*P. somniferum*	E	Turkey
PS08	Not specific on the label (physically: blue poppy seeds)	Unknown	No-E	Turkey
PS09	Wild opium poppy seeds (white)	*P. somniferum*	No-E	Spain
PS10	Wild poppy seeds	*P. rhoeas*	No-E	Spain
PS11	Not specific on the label (physically: blue poppy seeds)	Unknown	No-E	Czech Republic
PS12	Not specific on the label (physically: blue poppy seeds)	Unknown	E	No EU
PS13	Not specific on the label (physically: blue poppy seeds)	Unknown	E	No EU
MIX1	Sunflower, sesame, brown flax, pumpkin and poppy seeds	Unknown	No-E	Unknown
MIX2	Sunflower, sesame, brown flax, pumpkin and poppy seeds	Unknown	No-E	Unknown
MIX3	Pumpkin, sunflower, sesame, gold flax, brown flax and poppy seeds	Unknown	E	Unknown

The code of the samples: PS is because the samples are poppy seeds and MIX is the mixture of the different seeds. E/no-E: ecological/ no ecological cultivation, No EU: not from the European Union

Table S2. Optimal parameters of MRM for the analysis of each opium alkaloid by UHPLC-QqQ-MS/MS: precursor and fragment ions, cone voltage (CV), collision energy (CE) and retention time (Rt) of each opium alkaloid.

Analytes	Precursor ion (Q_1, m/z, $[M+H]^+$)	Fragment ion[a] (Q_3, m/z)	CE (eV)	Rt (min)
Morphine	286.6	**152.0**<br>165.0<br>201.0<br>229.0	55<br>35<br>20<br>20	1.556
Morphine-D3	289.0	**153.1**<br>157.0<br>165.0<br>201.0	20<br>35<br>35<br>35	1.554
Codeine	300.1	182.9<br>**198.9**<br>215.0<br>225.0	25<br>25<br>20<br>20	1.935
Oripavine	298.3	237.1<br>**249.1**<br>267.1	15<br>15<br>15	2.044
Thebaine	312.3	**58.1**<br>221.0<br>250.8<br>266.2	15<br>25<br>20<br>20	2.598
Papaverine	340.4	**202.0**<br>324.0	20<br>25	2.765
Noscapine	414.2	**220.0**<br>352.7	20<br>20	2.813

[a]The fragment ion used for the quantification are in bold

Table S3. Equations of adsorption kinetic and isotherm

$Q_e = \frac{(C_o - C_e)V}{W}$	Adsorption capacity	(1)
$\ln(q_e - q_t) = lnqe - k_1 t$	Lagergren's pseudo-first order	(2)
$\frac{t}{q_t} = \frac{1}{k_2 q_e^2} + \left[\frac{1}{q_e}\right] t$	Pseudo-second order	(3)
$q_t = k_p t^{1/2} + C$	Intra-particle diffusion	(4)
$\frac{1}{Q_e} = \frac{1}{Q_{max}} + \frac{1}{K_L Q_{max} C_e}$	Langmuir model	(5)
$Log\ q_e = logK_F + \frac{1}{n} logC_e$	Freundlich model	(6)

C_o and C_e: initial and equilibrium concentrations of the target analytes (μg/L), respectively; V: volume of the solution (L); W: mass of the adsorbent (g); k_1: pseudo-first order rate constant constant (min^{-1}); q_e and q_t: amounts of opium alkaloids adsorbed at equilibrium and time (mg/g), respectively; k_2: pseudo-second order adsorption rate constant (g/mg min); q_t: amount of opium alkaloids adsorbed at time t (mg/g); k_p: intraparticle diffusion rate (mg/L min^2); C: intercept; Q_{max}: maximum monolayer capacity of the adsorbent (mg/); K_L: Langmuir binding constant which is related to the energy of adsorption (L/mg); K_F: is the Freundlich constant (L/mg); n: is the heterogeneity factor (dimensionless).

Table S4. Kinetic parameters of the adsorption of six opioid alkaloids with 100 mg Fe_3O_4@SiO_2@mSiO_2 material for different times (1-20 min) based on different kinetic models.

		Pseudo-first order model			Pseudo-second order model			Intra-particle model		
	$Q_{e, exp}$ (mg/g)	R^2	K_1 (min^{-1})	$Q_{e, cal}$ (mg/g)	R^2	K_2 (g/mg min)	$Q_{e, cal}$ (mg/g)	R^2	K_p (mg/g min^2)	C
Morphine	13.23	0.0004	0.0042	1.05	0.9866	2925.58	17.04	0.9705	1.9732	7.33
Codeine	10.18	0.2047	-0.0864	0.10	0.9944	2472.37	11.68	0.7544	0.4352	9.29
Thebaine	11.61	0.0211	-0.0049	1.21	0.9976	7535.20	10.42	0.0594	-0.0670	10.48
Papaverine	13.74	0.0001	-0.0002	0.37	1.0000	162916.89	13.39	0.0093	-0.0040	13.37
Noscapine	13.91	0.7720	0.2132	0.21	0.9993	12749.19	13.97	0.5173	0.1454	13.08
Oripavine	13.02	0.7028	0.0449	0.04	0.9785	2004.18	15.27	0.9546	1.5342	7.28

$Q_{e, exp}$: amounts of opium alkaloids adsorbed at equilibrium, experimental; K_1: pseudo-first order rate constant constant; $Q_{e, cal}$: amounts of opium alkaloids adsorbed at equilibrium, calculated; K_2: pseudo-second order adsorption rate constant; K_p: intraparticle diffusion rate; C: intercept.

Table S5. Recovery values (%) ± standard deviation (SD) obtained in MSPE procedure with the solvents used in the SLE of poppy seeds at different desorption times (1, 5, 10 and 20 min).

Desorption time (min)	Desorption solvent	Morphine	Codeine	Tebaine	Papaverine	Noscapine	Oripavine
1	AcN/water/formic acid, 80/19/1	0.31 ± 0.22	32 ± 9	28 ± 9	47 ± 16	42 ± 14	0.71 ± 0.12
	MeOH/water 50/50	0.24 ± 0.11	22 ± 4	18 ± 3	22 ± 3	22 ± 3	0.52 ± 0.01
	MeOH 0.1% acetic acid	0.21 ± 0.04	12.81 ± 0.12	16.21 ± 0.31	26 ± 1	25.62 ± 0.82	0.34 ± 0.01
	MeOH	0.14 ± 0.02	10 ± 1	12.12 ± 0.72	23.03 ± 0.34	24 ± 1	0.33 ± 0.11
5	AcN/water/formic acid, 80/19/1	0.51 ± 0.31	45 ± 12	56 ± 4	67 ± 23	58 ± 18	1.21 ± 0.11
	MeOH/water 50/50	0.43 ± 0.32	35 ± 2	30.61 ± 0.21	42 ± 6	40 ± 3	0.71 ± 0.52
	MeOH 0.1% acetic acid	0.43 ± 0.13	18 ± 1	22.43 ± 0.63	38.51 ± 0.02	37.41 ± 0.52	0.71 ± 0.13
	MeOH	0.22 ± 0.03	11 ± 1	13.64 ± 0.72	29.22 ± 0.24	31 ± 1	0.74 ± 0.14
10	AcN/water/formic acid, 80/19/1	0.21 ± 0.01	62 ± 2	67 ± 3	93 ± 1	96.42 ± 0.23	0.72 ± 0.12
	MeOH/water 50/50	0.23 ± 0.12	21 ± 2	19 ± 2	13 ± 1	19 ± 2	0.44 ± 0.01
	MeOH 0.1% acetic acid	0.24 ± 0.12	12 ± 2	12.21 ± 0.61	15 ± 2	20 ± 2	0.52 ± 0.01
	MeOH	0.11 ± 0.03	9 ± 1	9 ± 1	11.03 ± 0.32	17 ± 1	0.33 ± 0.12
20	AcN/water/formic acid, 80/19/1	0.40 ± 0.13	79 ± 2	106 ± 26	121 ± 4	12 ± 3	1.52 ± 0.31
	MeOH/water 50/50	0.32 ± 0.14	39 ± 2	36 ± 2	28 ± 5	38 ± 2	0.93 ± 0.12
	MeOH 0.1% acetic acid	0.40 ± 0.12	16 ± 1	17.14 ± 0.72	22 ± 1	32 ± 1	1.25 ± 0.13
	MeOH	0.11 ± 0.03	11 ± 1	11.34 ± 0.83	14 ± 1	22 ± 2	0.33 ± 0.13

AcN: acetonitrile; MeOH: methanol.

Table S6. Recovery values (%) ± standard deviation (SD) obtained in MSPE procedure with eight acidified, non-modified or basified desorption solvents (acid with 1% formic acid, basic with 1% ammonia and basic with 10% ammonia).

pH	Desorption solvent	MOR	COD	THE	PAP	NOS	ORI
Non-modificated pH	Ether	-	10.61 ± 0.21	10.62 ± 0.12	14.01 ± 0.01	33.71 ± 0.51	0.71 ± 0.01
	DCM	-	2 ± 2	12.61 ± 0.41	77.01 ± 0.42	93 ± 1	-
	Chl	-	1.12 ± 0.11	11.21 ± 0.52	44.01 ± 0.53	47.41 ± 0.62	-
	Isopropanol	-	4.23 ± 0.23	4.64 ± 0.44	8.02 ± 0.42	13.92 ± 0.73	0.92 ± 0.02
	AcN	-	16 ± 3	16.21 ± 0.73	28.13 ± 0.71	29.51 ± 0.12	-
	MeOH	-	8.34 ± 0.32	9.40 ± 0.92	15.24 ± 0.92	18.02 ± 0.11	1.10 ± 0.03
	Water	-	7.12 ± 0.91	4.32 ± 0.41	2.91 ± 0.44	3.23 ± 0.42	0.74 ± 0.02
	EtOAc	-	2 ± 1	2.93 ± 0.32	22.30 ± 0.32	36.92 ± 0.84	-
Acid pH with 1% formic acid	Ether	-	0.4 ± 0.21	0.81 ± 0.11	1.50 ± 0.12	7.84 ± 0.12	-
	DCM	-	2.92 ± 0.51	38 ± 2	40.32 ± 0.21	51.83 ± 0.52	-
	Chl	-	1.51 ± 0.11	24 ± 1	26.73 ± 0.23	38.13 ± 0.21	-
	IPA	-	5.92 ± 0.31	5.21 ± 0.12	3.01 ± 0.90	6.14 ± 0.03	-
	AcN	-	29.64 ± 0.32	25.31 ± 0.33	31.71 ± 0.42	36.12 ± 0.04	-
	MeOH	-	25.93 ± 0.14	24.02 ± 0.44	21.01 ± 0.23	26.21 ± 0.12	-
	Water	-	11 ± 1	3.73 ± 0.41	2.01 ± 0.02	2.41 ± 0.32	-
	EtOAc	-	7 ± 1	12.91 ± 0.22	14.14 ± 0.11	29.61 ± 0.34	-
Basic pH with 1% ammonia	Ether	3.31 ± 0.12	48.81 ± 0.22	69.41 ± 0.21	73.31 ± 0.31	82.01 ± 0.04	16.72 ± 0.01
	DCM	4.24 ± 0.31	68.5 ± 1.11	67.42 ± 0.32	82.82 ± 0.43	75.72 ± 0.23	20.32 ± 0.42
	Chl	1.80 ± 0.33	23 ± 1	16.72 ± 0.31	17.72 ± 1.12	16.22 ± 0.03	5.22 ± 0.21
	IPA	1.81 ± 0.12	19.11 ± 0.21	20.71 ± 0.72	30.22 ± 0.83	31.41 ± 0.14	6.31 ± 0.31
	AcN	0.32 ± 0.04	45.41 ± 0.22	49.11 ± 0.43	62.54 ± 0.22	61.62 ± 0.04	2.11 ± 0.11
	MeOH	2.81 ± 0.01	44.43 ± 0.33	42.54 ± 0.04	55.64 ± 1.53	54.51 ± 0.32	9.51 ± 0.22
	Water	0.43 ± 0.11	10.30 ± 0.42	9.72 ± 0.11	24.93 ± 0.24	19.92 ± 0.42	1.91 ± 0.01
	EtOAc	1.41 ± 0.40	28 ± 1	29.60 ± 0.31	40.83 ± 0.51	39.51 ± 0.41	4.51 ± 0.52
Basic pH with 10% ammonia	Ether	3.22 ± 0.21	70 ± 2	98 ± 1	113.7 ± 0.21	115.72 ± 0.01	16.70 ± 0.22
	DCM	15.72 ± 0.32	85 ± 2	81.12 ± 0.21	99.62 ± 0.22	92.63 ± 0.02	22.60 ± 0.42
	Chl	3.81 ± 0.84	54 ± 1	60.12 ± 0.81	73.12 ± 0.04	57.64 ± 0.14	13.31 ± 0.33
	IPA	0.91 ± 0.02	12.64 ± 0.51	14.84 ± 0.33	23.24 ± 0.12	21.03 ± 0.13	1.71 ± 0.84
	AcN	3.81 ± 0.14	44.43 ± 0.32	51.04 ± 0.72	60.73 ± 0.32	57.22 ± 0.22	10.30 ± 0.12
	MeOH	3.21 ± 0.51	55.93 ± 0.81	62.52 ± 0.74	72.63 ± 0.33	68.73 ± 0.74	8.50 ± 0.03
	Water	2.61 ± 0.31	16.74 ± 0.22	20.92 ± 0.12	45.21 ± 0.14	29.44 ± 0.53	5.22 ± 0.33
	EtOAc	1.32 ± 0.01	17.21 ± 0.71	21.23 ± 0.33	22.50 ± 0.14	21.22 ± 0.42	4.32 ± 0.24

MOR: morphine; COD: codeine; THE: thebaine; PAP: papaverine; NOS: noscapine; ORI: oripavine; Ether: diethyl ether; DCM: dichlorometane; Chl: chloroform; IPA: isopropapol; AcN: acetonitrile; MeOH: methanol; EtOAc: ethyl acetate; -: no detected

Table S7. Recovery values (%) ± standard deviation (SD) obtained in MSPE procedure with 20 min adsorption with the mixture of the solvents that showed the best results in desorption step with different proportions (50/50, 80/20 and 20/80, v/v), all of them at basic pH with solvent/ammonia, 90/10 v/v.

Time (min)	Desorption solvent	MOR	COD	THE	PAP	NOS	ORI
20	Ether/AcN, 50/50	10.21 ± 0.21	63.91 ± 0.62	72.41 ± 0.64	63.62 ± 0.41	67 ± 3	21.82 ± 0.62
	Ether/AcN, 80/20	12 ± 1	74.61 ± 0.81	85.11 ± 0.24	91.81 ± 0.13	93.12 ± 0.12	29.62 ± 0.43
	Ether/AcN, 20/80	6.71 ± 0.42	51 ± 1	50.81 ± 0.04	66.02 ± 0.24	63.21 ± 0.23	13.51 ± 0.21
	DCM/AcN, 50/50	7.54 ± 0.23	67 ± 1	72.34 ± 0.12	72.54 ± 0.71	66.12 ± 0.64	24.12 ± 0.32
	DCM/AcN, 80/20	5.01 ± 0.22	55.84 ± 0.62	56.33 ± 0.23	59.12 ± 0.52	53.24 ± 0.42	19.93 ± 0.44
	DCM/AcN, 20/80	3.92 ± 0.01	38.31 ± 0.74	42.91 ± 0.32	42.96 ± 0.44	40.11 ± 0.51	13.71 ± 0.42
	Ether/MeOH, 50/50	10.23 ± 0.84	37.08 ± 0.83	38.52 ± 0.32	43 ± 1	49.04 ± 0.54	11.52 ± 0.13
	Ether/MeOH, 80/20	20.74 ± 0.82	74.12 ± 0.72	79.71 ± 0.21	102.71 ± 0.01	106.62 ± 0.42	24.11 ± 0.22
	Ether/MeOH, 20/80	7.52 ± 1.13	33.81 ± 0.52	32.80 ± 0.53	43.14 ± 0.12	44.23 ± 0.21	9.93 ± 0.21
	DCM/MeOH, 50/50	8.34 ± 0.84	41.01 ± 0.54	46.40 ± 0.42	58.64 ± 0.34	56.62 ± 0.14	10.12 ± 0.14
	DCM/MeOH, 80/20	8.52 ± 0.52	82.61 ± 0.33	86.61 ± 0.34	112.92 ± 0.11	109.44 ± 0.22	22.71 ± 0.52
	DCM/MeOH, 20/80	11.22 ± 0.43	40.43 ± 0.12	42.70 ± 0.23	53.20 ± 0.43	53.71 ± 0.21	12.22 ± 0.91
40	Ether/AcN, 50/50	11.31 ± 0.22	82.22 ± 0.34	90.83 ± 0.82	82.41 ± 0.14	84.10 ± 0.14	25 ± 1
	Ether/AcN, 80/20	14.42 ± 0.34	94.51 ± 0.32	102.61 ± 0.91	112.64 ± 0.02	105.81 ± 0.02	38 ± 2
	Ether/AcN, 20/80	7.34 ± 0.22	59.31 ± 0.23	58.64 ± 1.12	75.43 ± 0.21	71.52 ± 0.35	15.24 ± 0.51
	DCM/AcN, 50/50	9.02 ± 0.83	75.22 ± 0.83	80.32 ± 0.33	79.54 ± 0.42	72.30 ± 0.57	27.81 ± 0.62
	DCM/AcN, 80/20	6.71 ± 0.82	59.31 ± 0.79	60.54 ± 0.42	64.01 ± 0.14	58.31 ± 0.54	21.92 ± 0.84
	DCM/AcN, 20/80	4.52 ± 1.14	64.24 ± 0.48	69.22 ± 0.54	68.64 ± 0.83	63.52 ± 0.65	16.90 ± 0.91
	Ether/MeOH, 50/50	11.71 ± 0.82	59.85 ± 0.19	61.11 ± 0.63	74 ± 1	75.73 ± 0.37	14.50 ± 0.54
	Ether/MeOH, 80/20	33.84 ± 0.46	105.83 ± 0.24	109.93 ± 0.52	146 ± 1	142.20 ± 0.24	36 ± 2
	Ether/MeOH, 20/80	11.33 ± 0.19	65.45 ± 0.12	59.70 ± 0.41	75.53 ± 0.74	78.01 ± 0.11	13 ± 1
	DCM/MeOH, 50/50	10.10 ± 0.26	54.91 ± 0.51	61.01 ± 0.82	76.42 ± 0.24	72.72 ± 0.62	12.80 ± 0.12
	DCM/MeOH, 80/20	12.12 ± 0.43	96.64 ± 0.22	98.90 ± 0.43	130.33 ± 0.12	125 ± 4	26.94 ± 0.82
	DCM/MeOH, 20/80	13.42 ± 0.53	60.44 ± 0.11	63.10 ± 0.32	78.44 ± 0.14	78 ± 2	14.7 ± 0.71
60	Ether/AcN, 50/50	11 ± 1	86 ± 1	95.34 ± 0.41	86.22 ± 0.52	87.61 ± 0.32	25.61 ± 0.42
	Ether/AcN, 80/20	14 ± 1	96.65 ± 0.89	103.41 ± 0.82	114.04 ± 0.31	106 ± 1	39.01 ± 0.11
	Ether/AcN, 20/80	7.32 ± 0.36	60 ± 2	59.93 ± 0.44	76.74 ± 0.32	72.62 ± 0.98	15.84 ± 0.22
	DCM/AcN, 50/50	9.01 ± 0.12	76.04 ± 0.34	81.14 ± 0.32	80.72 ± 0.44	73.31 ± 0.24	28.41 ± 0.44
	DCM/AcN, 80/20	6.71 ± 0.67	59.82 ± 0.87	61.11 ± 0.13	64.83 ± 0.83	59.42 ± 0.35	22.44 ± 0.63
	DCM/AcN, 20/80	4.51 ± 0.34	67.52 ± 0.65	72.64 ± 0.01	72.44 ± 0.42	67 ± 4	17.30 ± 0.82
	Ether/MeOH, 50/50	12 ± 1	11.64 ± 0.15	72.21 ± 0.44	90.32 ± 0.21	89.32 ± 0.12	15.21 ± 0.44
	Ether/MeOH, 80/20	35.64 ± 0.12	114.53 ± 0.35	114.22 ± 0.42	153.31 ± 0.14	146.21 ± 0.85	37.24 ± 0.72
	Ether/MeOH, 20/80	13.22 ± 0.34	89 ± 1	76.80 ± 0.41	96.04 ± 0.02	98.12 ± 0.67	14.74 ± 0.81
	DCM/MeOH, 50/50	10.70 ± 0.96	65.64 ± 0.96	70.44 ± 0.34	88 ± 1	83.44 ± 0.54	14.01 ± 0.43
	DCM/MeOH, 80/20	12.71 ± 0.68	121 ± 3	100.10 ± 0.83	132.42 ± 0.14	127 ± 2	27.21 ± 0.63
	DCM/MeOH, 20/80	13.50 ± 0.18	62 ± 2	67.73 ± 0.74	84 ± 2	83.23 ± 0.48	15.01 ± 0.42

MOR: morphine; COD: codeine; THE: thebaine; PAP: papaverine; NOS: noscapine; ORI: oripavine; Ether: diethyl ether; DCM: dichlorometane; AcN: acetonitrile; MeOH: methanol.

Table S8. Recovery values (%) ± standard deviation (SD) obtained in MSPE procedure with 1 min adsorption after different number of consecutive desorptions with diethyl ether/methanol, 80/20, v/v, with 10% ammonia at 1 min.

Number of consecutive desorptions	Morfina	Codeina	Tebaina	Papaverina	Noscapina	Oripavina
1	34.69 ± 0.39	58.12 ± 0.03	78.51 ± 0.12	78.92 ± 0.52	45.82 ± 0.02	36.21 ± 0.33
2	44.42 ± 0.51	72.81 ± 0.89	97.42 ± 0.13	99.71 ± 0.11	66.41 ± 0.11	45.24 ± 0.22
3	46.81 ± 0.08	77.02 ± 0.48	102.52 ± 0.18	105.42 ± 0.33	72.04 ± 0.12	46.63 ± 0.14
4	47.44 ± 0.22	77.58 ± 0.78	103.18 ± 0.57	106.48 ± 0.22	73.10 ± 0.04	46.92 ± 0.21
5	47.57 ± 0.14	78.37 ± 0.41	104.07 ± 0.12	109.34 ± 0.64	76.01 ± 0.12	47.11 ± 0.19

Table S9. Instrumental validation parameters (solvent linearity, solvent calibration, LOQ and LOD).

Analytes	Solvent linearity (μg/L)	Solvent calibration (R^2)	LOQ (μg/L)	LOD (μg/L)
Morphine	0.1-5000	y = 5090x + 102761 (0.999)	8 x 10^{-2}	2 x 10^{-2}
Codeine	0.1-5000	y = 2986x + 451315 (0.999)	9 x 10^{-2}	3 x 10^{-2}
Thebaine	0.01-5000	y = 9235x + 905583 (0.999)	5 x 10^{-3}	1 x 10^{-3}
Papaverine	0.01-5000	y = 41638x + 2235050 (0.999)	5 x 10^{-3}	1 x 10^{-3}
Noscapine	0.01-5000	y = 66219x + 4092136 (0.999)	9 x 10^{-3}	3 x 10^{-3}
Oripavine	0.1-5000	y = 3531x + 204673 (0.999)	6 x 10^{-2}	2 x 10^{-2}

LOQ: limit of quantification; LOD: limit of detection.
